# A Comparative Quantitative Assessment of Axonal and Dendritic mRNA Transport in Maturing Hippocampal Neurons

**DOI:** 10.1371/journal.pone.0065917

**Published:** 2013-07-22

**Authors:** Gunja K. Pathak, James M. Love, Joshua Chetta, Sameer B. Shah

**Affiliations:** 1 Fischell Department of Bioengineering, University of Maryland, College Park, Maryland, United States of America; 2 Departments of Orthopaedic Surgery and Bioengineering, University of California San Diego, La Jolla, California, United States of America; International Centre for Genetic Engineering and Biotechnology, Italy

## Abstract

Translation of mRNA in axons and dendrites enables a rapid supply of proteins to specific sites of localization within the neuron. Distinct mRNA-containing cargoes, including granules and mitochondrial mRNA, are transported within neuronal projections. The distributions of these cargoes appear to change during neuronal development, but details on the dynamics of mRNA transport during these transitions remain to be elucidated. For this study, we have developed imaging and image processing methods to quantify several transport parameters that can define the dynamics of RNA transport and localization. Using these methods, we characterized the transport of mitochondrial and non-mitochondrial mRNA in differentiated axons and dendrites of cultured hippocampal neurons varying in developmental maturity. Our results suggest differences in the transport profiles of mitochondrial and non-mitochondrial mRNA, and differences in transport parameters at different time points, and between axons and dendrites. Furthermore, within the non-mitochondrial mRNA pool, we observed two distinct populations that differed in their fluorescence intensity and velocity. The net axonal velocity of the brighter pool was highest at day 7 (0.002±0.001 µm/s, mean ± SEM), raising the possibility of a presynaptic requirement for mRNA during early stages of synapse formation. In contrast, the net dendritic velocity of the brighter pool increased steadily as neurons matured, with a significant difference between day 12 (0.0013±0.0006 µm/s ) and day 4 (−0.003±0.001 µm/s) suggesting a postsynaptic role for mRNAs in more mature neurons. The dim population showed similar trends, though velocities were two orders of magnitude higher than of the bright particles. This study provides a baseline for further studies on mRNA transport, and has important implications for the regulation of neuronal plasticity during neuronal development and in response to neuronal injury.

## Introduction

The geometry and unusual polarity of neurons imposes a tremendous challenge on biological communication between the cell body and neuronal projections such as axons and dendrites. One such challenge is the appropriate localization of translated proteins, which can vary depending on intracellular and extracellular cues [1]. A large fraction of protein deployment within neuronal projections occurs through the active or passive transport of proteins synthesized in the cell body [2]. Alternately, increasing evidence suggests that proteins may be translated locally, along an axon or dendrite or at their termini [3], [4], [5], [6], [7], [8]. Though synthesis may occur within neurites, mRNA must still be transported, often as part of a granular complex containing additional translational and regulatory machinery, from the cell body to sites of local translation. Local synthesis has been linked to neuronal development, survival, and learning and memory [9]. Specific activities playing a role in these processes include axon guidance, synapse formation and synaptic refinement [10]. Clinically, defects in local mRNA regulation have been linked to several neurological disorders, including fragile X syndrome and spinal muscular atrophy [11].

Previous work on mechanisms and regulation of local protein synthesis has been performed in both the peripheral nervous system (PNS) and central nervous system (CNS). In the PNS, evidence for local protein synthesis was presented as early as 1970, when ribosome-like particles were visualized in axons by electron microscopy (EM) [6], [7], [8]. Since then, studies have confirmed the identity of peripheral ribosomal domains within myelinated mammalian axons [10], [12], and gene-profiling studies have revealed many different proteins that can be synthesized locally in axons [3], [4], [5]. Regulation of the transport of mRNA transcripts corresponding to these proteins is also essential for local synthesis. Recent work suggests that mRNA transport is tightly coupled to the activation of local protein synthetic pathways, possibly through signaling pathways initiated by growth factors [4], [13]. These regulatory pathways have also been studied in models of axonal injury, recovery from which necessitates increased local protein synthesis [4], [14], [15]. Of particular interest are RNA binding proteins (RBP) such as Staufen and fragile X mental retardation protein (FMRP), which regulate the distribution of mRNA.

In the CNS, compelling functional roles for local protein synthesis have been identified in development as well as the regulation of synaptic stabilization, long-term potentiation or depression (LTP or LTD), and the consolidation of memory. Translational profiling and localization studies suggest that hundreds of different proteins can be locally synthesized in the axons [3] and dendrites [16]. In addition, multiple components of translational machinery, such as mRNA, signal recognition particles (SRP), ER proteins, and Golgi components have also been observed in the axons [17] and in dendrites [5], [6]. Developmentally, dendritic protein synthesis increases during synaptogenesis and decreases into adulthood [11], [18]. As in the PNS, several RBP have been implicated in coupling the transport of mRNA into axons and dendrites with local protein synthesis [11], [19].

Collectively, studies in both the PNS and CNS reveal multiple conceptual similarities in hypothesized mechanisms guiding the coupling of mRNA transport and translation. However, a significant gap in our understanding of this coupling persists, in part due to a lack of rigorous criteria by which transport may be assessed and compared. As a first step towards filling this gap, we have developed methods to quantify several parameters that describe the dynamics of mRNA transport and localization. Using these methods, we compared mRNA transport in the axons and dendrites of cultured hippocampal neurons at various stages of neurite outgrowth and developmental maturity. We have validated these methods by comparing mRNA transport to that of mitochondria, a well-characterized transport cargo. Our results provide a baseline for future studies on mRNA transport, and raise interesting hypotheses regarding the plasticity of transport during hippocampal development.

## Results

### Localization of RNA granules in maturing hippocampal neurites

**Figure 1:**
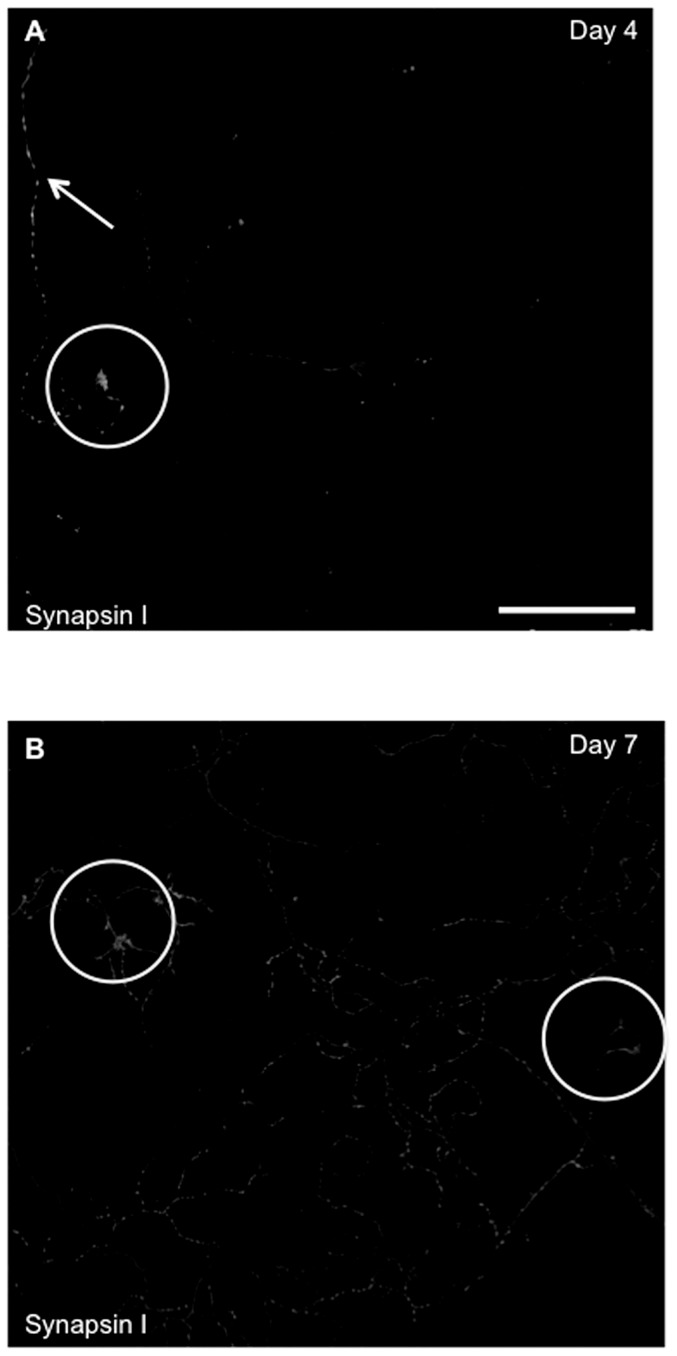
Maturity of hippocampal neurons at different days in culture. The synaptic vesicle protein Synapsin I is a marker for the maturity of the hippocampal neurons [20]. Immunolabeling with Synapsin I at day 4 (A) shows granular proteins concentrated in the distal axon and growth cone, as there is no cell-cell contact (arrow – protein in distal axon, circle- protein in growth cone), while there is a considerable increase in fluorescence intensity at day 7 (B). There is formation of large cluster of vesicles at sites of synaptic contact at day 7 (Circles-shows cell connection). The images illustrated here have been converted to grayscale and contrast enhanced, to emphasize neurites. Bar is 50 µm.

Immunofluorescence and DIC imaging were performed to examine mRNA localization at different phases of developmental maturity and within hippocampal neurites of different polarity. At days 4, 7, and 12, several criteria were used to determine the developmental phase of the neurons and to assess polarity. First, we tested whether a neurite contacted another cell. At day 4, the majority of neurites displayed a free growth cone, while at days 7 and 12, the termini of most neurites intersected with other cells. At day 12, there was also significantly more axonal branching per neuron compared to day 7 (9.0±1.3 vs. 2.5±1.08; Mean ± SEM; t-test p<0.04), though the total number of projections and dendritic branching per neuron was unchanged. Second, the synaptic vesicle protein synapsin I was labeled to determine synaptic maturity [20], [21]. At day 4, as expected based on previous work [23], synapsin localized primarily as small puncta along neurites and as larger densities at terminal growth cones (Fig. 1a). By day 7, larger densities of synapsin I were observed, with accumulations of vesicles at sites of neurite contact indicating the presence of stable synapses (Fig. 1b).

**Figure 2:**
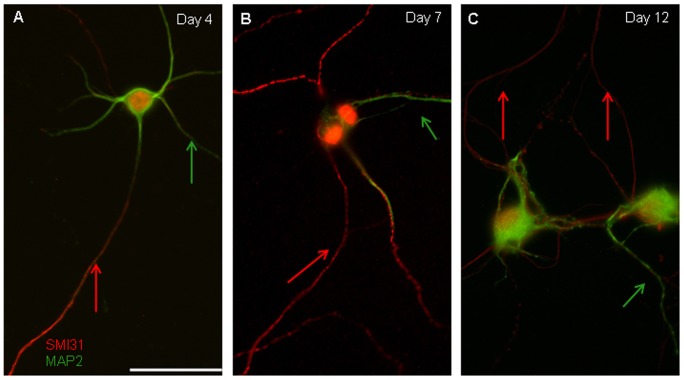
Hippocampal neurons are differentiated and present distinct morphology for both the axon and dendrites. Dendrites have shorter process and taper more gradually (arrows), while axons display a long, narrow process with minimal tapering (arrows in all figures). (A–C) Expression of phosphorylated neurofilaments SMI-31 (red) and microtubule-associated protein MAP2 (green) (A) Double-label immunofluorescence of SMI-31 (red) and MAP2 (green) at 4 DIV (days *in vitro*). Dendrites (MAP2) are shorter with gradual tapering projections whereas axon (SMI-31) stain display long narrow processes. (B) Double-label immunofluorescence of SMI-31 (red) and MAP2 (green) at 7 DIV. (C) Double-label immunofluorescence of SMI-31 (red) and MAP2 (green) at 12 DIV. Bar is 50 µm.

To differentiate between dendrites and axons, we examined the geometry of neurites under DIC and fluorescence imaging. As previously described [22], [23], [24], axons exhibited a long, narrow process that emerged from the cell body with minimal tapering and dendrites displayed a shorter process that tapered more gradually as it emerged from the cell body (Fig. 2). We confirmed morphological assessments with immunofluorescence; axons were identified through the labeling of phosphorylated neurofilaments (SMI-31) and dendrites by MAP2 (Fig. 2). Spatially distinct SMI-31 and MAP2 labeling was apparent as early as day 3 (data not shown). However, the fluorescence pattern was more continuous within each neurite at days 4, 7, and 12 (Fig. 2a–c), confirming that neurons had fully differentiated at our earliest time point. Based on these criteria, then, neurons at day 4 with a free terminal were designated as immature (growing), differentiated neurons. Neurons at days 7 and 12 were both designated as differentiated neurons with stable synapses, with those at day 12 presumably more mature.

**Figure 3:**
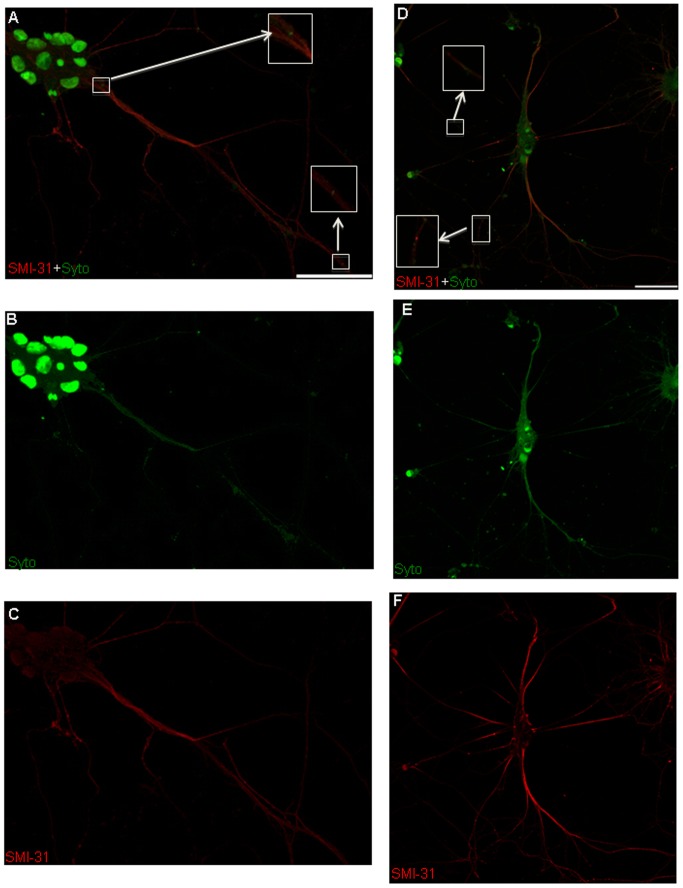
Confocal microscopy of mRNA localization in axons. (A,D) Double-label immunofluorescence shows Syto labeling in cell bodies (nuclear) and co-localization of mRNA (Syto, green) with an axonal marker (SMI-31, red). Green puncta (Syto) that are not co-labeled correspond to localization within dendrites. (Inset – expanded for more clear visualization). (B, E) Immunofluorescence of mRNA (Syto, green). (C,F) Immunofluorescence of axonal marker only (SMI-31, red). Bar is 50 µm.

**Figure 4:**
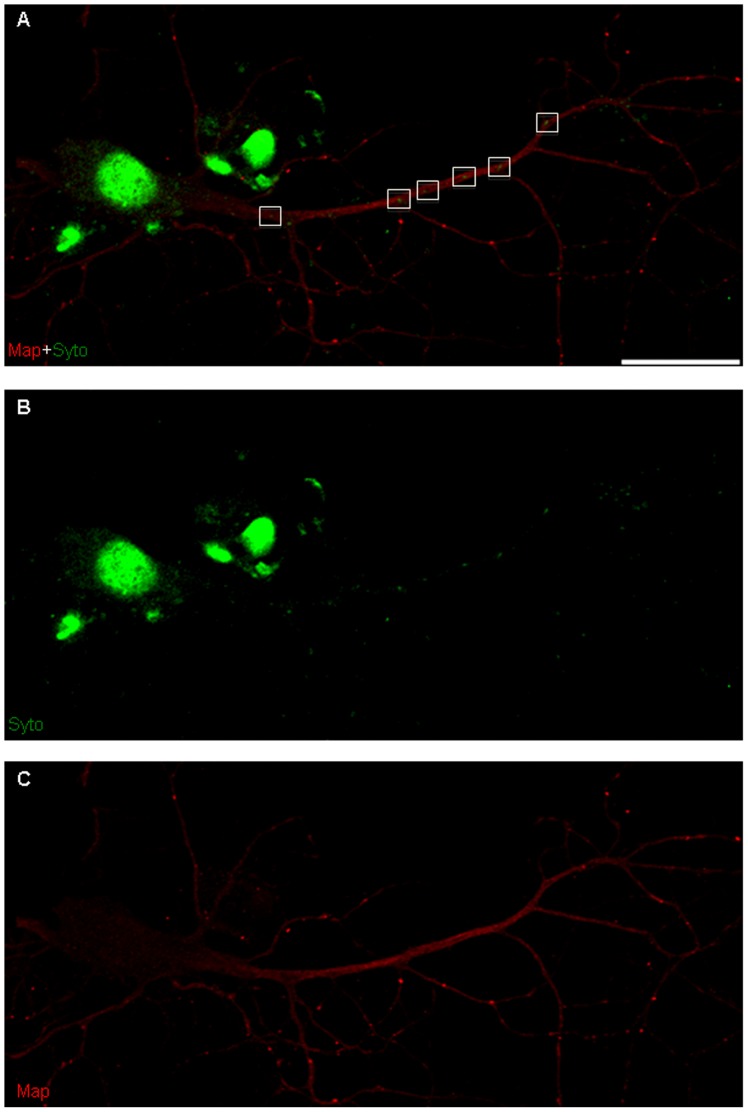
Confocal microscopy of mRNA localization in dendrites. (A) Double-label immunofluorescence shows Syto labeling in cell bodies (nuclear) and co-localization of mRNA (Syto, green) with a dendritic marker (MAP2, red). Green puncta (Syto) that are not co-labeled correspond to localization within axons. (B) Immunofluorescence of mRNA (Syto, green). (C) Immunofluorescence of dendritic marker (MAP2, red). Bar is 50 µm.

**Figure 5:**
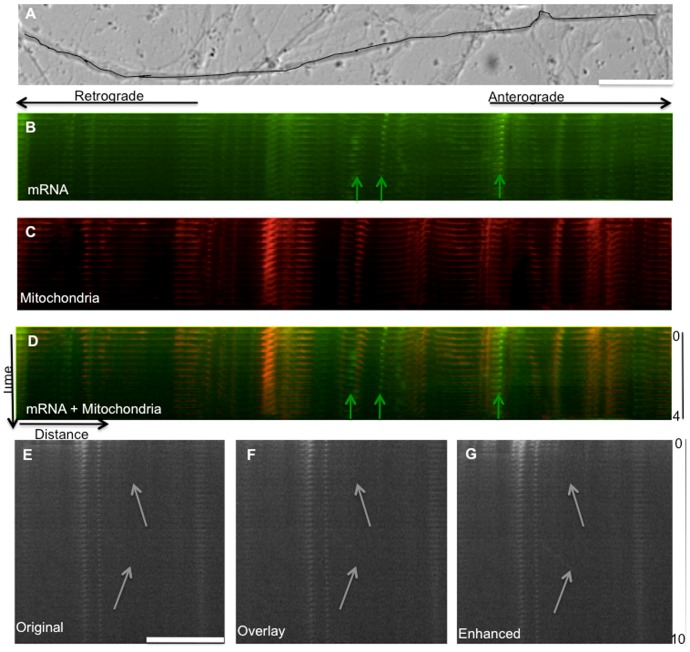
Methods of image analysis. Time-lapse images of neurites were taken under DIC and fluorescence conditions. After classifying the neurite as an axon or dendrite using the DIC image, RNA particles (Syto, green) and mitochondrial particles (Mitotracker, red) were fluorescently labeled. (A) DIC image of a neurite (B) Both mitochondrial (B) and non-mitochondrial RNA and (C) non-mitochondrial kymographs were generated along the length of the neurite visible in the imaging field. Particles that were present in both of the kymographs were concluded to be mitochondrial mRNA whereas particles that were only present in the non-mitochondrial RNA kymograph were considered mRNA particles (arrows). (D) Overlay of mRNA kymograph (green) and mitochondrial kymograph (red). (E) mRNA kymograph without contrast enhancement. (F) Kymograph from (E) following iterative overlay of 50% transparent image to visualize dim moving particles. (G) Kymograph from (E) following contrast enhancement to visualize dim mRNA moving particles. Additional details on enhancement of dim particles are shown in Figure S1. Bar is 20 µm.

To ascertain the localization of axonal and dendritic mRNA particles at each stage of development, cells were labeled with Syto, a nucleic acid stain that labels RNA in both intra- and extra-nuclear compartments of the cell [25], [26]. Syto co-localized with nuclei labeled with Hoechst dye and also localized to neurites (Fig. S1). Morphological assessment under DIC imaging conditions and counterstaining with SMI-31 and MAP2 antibodies revealed that RNA localized to both axons and dendrites (Fig. 3,4). To differentiate non-mitochondrial RNA from mitochondrial mRNA in these processes, neurons were co-labeled with Syto and MitoTracker. RNA puncta that did not co-localize with MitoTracker within a neurite were designated as non-mitochondrial particles of mRNA (Fig. 5b–d). The likelihood of a particle being mistakenly labeled as non-mitochondrial (i.e., a false positive) was low, given the high affinity and fluorescence intensity of MitoTracker. Subsequent kymograph analysis was performed on labeled non-mitochondrial mRNA (referred to from now as mRNA) and mitochondria to distinguish between the movements of these two spatially and functionally distinct cargoes (Fig. 5b–d).

RNase treatment was performed to test whether “dim” particles labeled with Syto were mRNAs (Fig. S2). Bright and dim mRNA particles were observed in kymographs of Syto, captured for only 5 minutes to minimize bleaching artifacts. Ribonuclease A treatment resulted in complete deletion of Syto signal in the neurites, and a strong suppression of signal in the cell body. Post-RNase, no puncta along the axon were as intense as the dimmest visible particle pre-RNase, suggesting that Syto indeed labeled both bright and dim mRNA particles (Fig. S2b–e).

### mRNA transport in dendrites and axons

Initial inspection of kymographs indicated 20–30 total fluorescent particles in each axon, with about half associated with mitochondria and half unassociated. Qualitatively, these particles appeared to move bidirectionally over short distances at slow rates. Closer inspection after the contrast enhancement of kymographs revealed another population of particles with very low fluorescence (Fig. 5e–g). These particles were difficult to find in individual frames of time-lapse movies as well as non-enhanced kymographs; therefore, we applied a sequence of image processing algorithms to enhance their contrast and confirm their presence (Fig. 5e–g, Fig. S3 and S4). These particles appeared more mobile than their bright counterparts, and did not co-localize with mitochondria. Given the possibility that these visually distinct pools of mRNA, designated as “bright” and “dim,” could have different functional roles, we analyzed their transport profiles separately rather than pooling their data.

**Figure 6:**
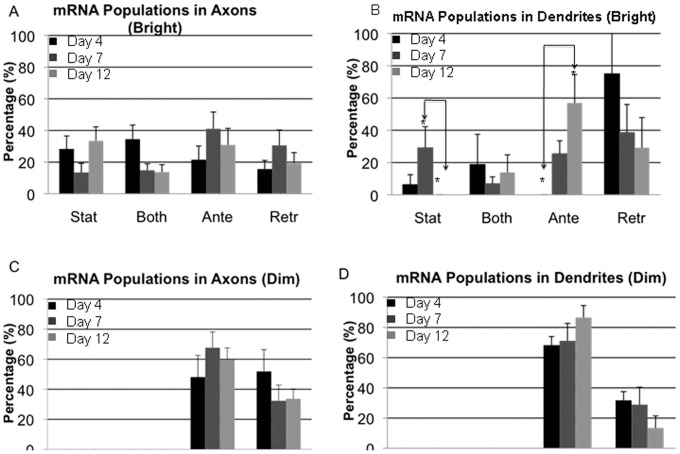
Net directionality of particle movement over its lifetime. mRNA and mitochondrial particles were categorized as either moving or stationary. They were considered moving if their average velocity in either direction was greater than 0.001 µm/sec (0.1 mm/day). Particles that did not meet this criterion were designated stationary. (A) percent of bright mRNA particles in axons in each state for days 4, 7, and 12. (B) Percentage of bright mRNA particles in dendrites in each state for days 4, 7, and 12. There are significantly more moving particles in anterograde direction at day 12 compared to day 4 p<0.02 (ANOVA:Tukey). In contrast, there are significantly more stationary particles at Day 7 compared to Day 12 p<0.05 (ANOVA:Tukey). (C) Percentage of dim mRNA particles in axons in each state for days 4, 7, and 12. (D) Percentage of dim mRNA particles in dendrites in each state for days 4, 7, and 12.

**Figure 7:**
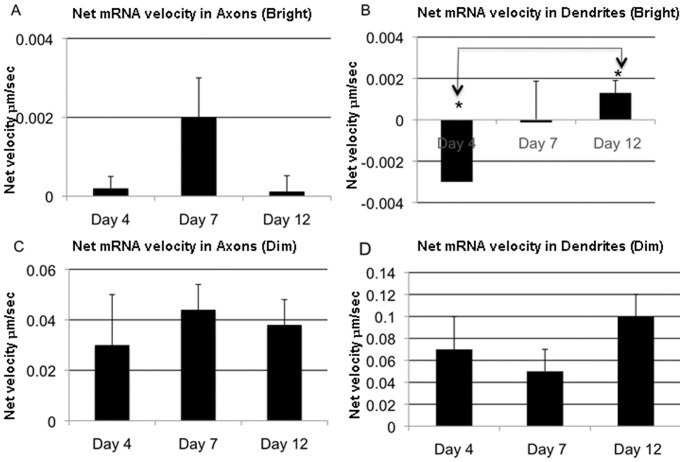
Average net velocity of individual dim and bright mRNAs particles in axons and dendrites. (A) Average of net bright mRNA velocity moving through axons. (B) Average of net bright mRNA velocity moving through dendrites. The net velocity is higher at day 12 compared to day 4 p<0.05 (ANOVA:Tukey). (C) Average of net dim mRNA velocity moving through axons. (D) Average of net dim mRNA velocity moving through dendrites. Values represent means ± SEM.

A detailed assessment of transport involved the extraction of several parameters, which are summarized in Table 1. The comparison of pooled particles of a particular identity (directionality and net velocity) is presented in Fig. 6 and Fig. 7. These parameters are attained from comprehensive raw data that describe the movement of individual particles (particle velocity and duration). For convenience, Tables 2 and 3 summarize results for all parameters and may serve as a useful roadmap through the extensive datasets. Raw data and their statistical comparisons are also presented as supplementary figures and tables (Fig. S5, S6, Table S1, S2, S3, S4).

**Table 1:** Summary of measured parameters used to describe transport profiles for individual particles or groups of particles.

*Parameter*	*Definition*
Maximum Velocity	The maximum velocity achieved during the lifetime of a particle in each direction
Average Velocity	The average velocity during the lifetime of a particle in each direction
Duration	The amount of time spent by a particle moving in each direction during its lifetime
Directionality	The net direction that a given particle moves over its lifetime within a neurite
Net Velocity	The overall directionality and velocity of individual particles

Detailed definitions are found in the Methods section.

**Table 2:** Summary of statistically significant differences in populations and net velocities for various classes of labeled cargoes.

Cargo	Population Analysis	Net Velocity
mRNA in Dendrites (Dim)	-	day 4 vs. ↑day 12
mRNA in Axons (Bright)	-	-
mRNA in Dendrites (Bright)	↑day 7 vs. day 12 (stat)day 4 vs. ↑day 12 (Ante)	-
Mitochondria in Axons	day 4 vs. ↑day 7 (Ante)	-
Mitochondria in Dendrites	-	day 4 vs. ↑day 7day 4 vs. ↑day 12

Dash indicates no significant difference for a given parameter. Raw data are provided in supplementary figures and tables.

**Table 3:** Summary of statistically significant differences in maximum velocities and track durations for various classes of labeled cargoes.

Cargo	Maximum Track Velocity	Track Duration
mRNA Axon anterograde (Dim)	-	-
mRNA Axon retrograde (Dim)	-	day 4 vs. ↑day 12↑day 7 vs. day 12
mRNA Dendrite anterograde (Dim)	day 7 vs. ↑day 12	-
mRNA Dendrite retrograde (Dim)	-	-
mRNA Axon anterograde (Bright)	↑day 7 vs. day 12	-
mRNA Axon retrograde (Bright)	day 4 vs. ↑day 7	-
mRNA Dendrite anterograde (Bright)	day 4 vs. ↑day 7day 4 vs. ↑day 12↑day 7 vs. day 12	-
mRNA Dendrite retrograde (Bright)	-	-
Mitochondria Axon anterograde	day 4 vs. ↑day 12↑day 7 vs. day 12	day 4 vs. ↑day 12day 7 vs. ↑day 12
Mitochondria Axon retrograde	↑day 4 vs. day 12↑day 7 vs. day 12	↑day 4 vs. day 12↑day 7 vs. day 12
Mitochondria Axon anterograde	N/A	N/A
Mitochondria Dendrite anterograde	day 4 vs. ↑day 7day 4 vs. ↑day 12↑day 7 vs. day 12	-
Mitochondria Dendrite retrograde	↑day 4 vs. day 12	↑day 4 vs. day 12

Dash indicates no significant difference for a given parameter. Raw data are provided in supplementary figures and tables.

#### Maximum and average velocity

Maximum and average directional velocities of mRNA particles in axons and dendrites were measured at different stages of neuronal maturity. For bright mRNA particles, two-way ANOVA indicated no effect of the type of neurite, no effect of stage of maturity and no interaction effect on maximum or average anterograde velocity; however, significant effects of the type of neurite (p<0.005), stage of maturity (p<0.01), and their interaction (p<0.0001) were observed on maximum and average retrograde velocities (Table S1).

Several interesting features emerged when distributions of velocities were compared. (Similar conclusions were obtained for both maximum and average velocities; for clarity, we have only shown figures for distributions of maximum velocities). In axons, bright mRNA particles showed significant differences in the distribution of maximum velocities in the anterograde direction between days 7 and 12 (p<0.002) and in the retrograde direction between days 4 and 7 (p<0.03; Fig. S5a–b). Anterograde particles in dendrites also showed a significant difference in the maximum velocity distributions at day 12 compared to day 4 (Fig. S5c–d), associated with a leftward shift of the distribution (decreased velocities). As suggested by the strong rightward shift in their velocity distribution, dim particles were faster than bright particles (p<0.0001, Fig. S5a–d). Two-way ANOVA revealed a significant effect of the type of neurite (p<0.0013), and stage of maturity (p<0.03), but no interaction effect on dim anterograde mRNA velocity. There was no significant effect of type of neurite, stage of maturity, or their interaction on dim retrograde mRNA velocity. Comparisons of distributions of velocities also revealed no significant differences.

#### Directionality analysis

To generate additional perspective on the overall movement patterns of individual mRNA particles, they were classified into anterogradely moving, retrogradely moving, bidirectionally moving, or stationary populations. Within axons, most bright mRNA particles were mobile (at least 75% at all days). Concurrent with a decrease in the proportion of stationary particles, the proportion of directionally moving particles (either anterograde or retrograde) significantly increased between day 4 and day 7 (∼35% vs. ∼70%) before decreasing again to an intermediate value at day 12 (∼50%). Though movement reflected an anterograde bias at all three days (Fig. 6a), there were no significant differences in anterograde or retrograde populations across days. This general trend was mirrored in the dim pool of particles as well (Fig. 6c). In this case, increases in the anterograde population between days 4 and 7 were balanced by a corresponding decrease in the retrograde pool.

In dendrites, most bright particles were also classified as mobile, with all particles moving by day 12 (Fig. 6b). This correlated with significant differences in stationary mRNA particles detected between days 7 and 12. Significant differences in directionality were also observed with increasing dendritic maturity. The proportion of particles moving anterogradely increased steadily with time, with nonsignificant differences being detected between days 4 and 7 (Fig. 6b; 0% and 25%, respectively) and significant differences being detected between days 4 day 12 (57%). In contrast, the proportion of particles moving retrogradely decreased steadily over time, though no significant differences were detected between any days. The anterograde bias was more pronounced in the dim pool of mRNA particles (Fig. 6d), with ∼70% of the particles moving anterogradely at days 4 and 7, and ∼85% moving anterogradely at day 12.

#### Duration of directional movements

We also calculated the percentage of time that mRNA and mitochondria spent moving in each direction within axons and dendrites at each time point. Two-way ANOVA of dim mRNA particles detected effects of the type of neurite (p<0.009), but no effect of maturity or interaction on anterograde and retrograde durations. For bright mRNA particles, analysis of distributions indicated a trend towards longer durations in both anterograde and retrograde directions at later days in both axons and dendrites, though there were no significant differences in the distributions of the durations in either direction (Fig. S6a–d). In contrast, dim particles did not show any trends towards increased duration at later days in axons or dendrites. In fact, anterograde durations in dendrites were actually slower at days 7 and 12 compared to day 4, with significant differences between day 4 and 7 (p<0.03), and day 7 and 12 (p<0.04) in the retrograde direction.

#### Net velocity

In order to extrapolate transport characteristics of individual mRNA particles to bulk transport at different stages of axonal and dendritic development, we estimated net velocities for specific classes of particles at a given stage of maturity. Net velocity calculations combined the individual characteristics of each particle into an aggregate measure of directional transport. Several interesting trends were observed. The net velocity of bright mRNA within axons was highest at day 7, at early stages of synapse maturity, but decreased sharply at day 12 (Fig. 7a). In contrast, net velocity for bright particles moving within dendrites continued to increase through day 12, with significant difference between day 4 and day 12 (p<0.05, Fig. 7b). Net velocity attributed to dim mRNA particles revealed similar trends in axons; however, the velocities were two orders of magnitude higher (Fig. 7c). In dendrites, net velocity was always positive, and experienced a slight decline at day 7 before rebounding at day 12 (Fig. 7d).

To investigate the relative contributions of individual parameters to net velocity, and thus perhaps glean some insight into regulatory mechanisms, we performed multiple linear regression analysis, with net velocity as the dependent variable and velocity and duration as independent variables. As reflected by their higher beta weights, velocities were for each group predominantly, though not exclusively, the dominant independent variable (Table 4). However, r^2^ values were surprisingly low, given that the independent variables reflected parameters used to calculate net velocity (Table 4). This perhaps implies the importance of relative proportions of directionally moving particles, which could not be captured in regression analysis.

**Table 4:** Results comparing beta coefficients.

Experiment	Variable	Coefficient	R-squared	variable	coeff
Mitochondria Axon	**Velocity**	0.18	0.04	duration	−0.047
Mitochondria Dendrites	**Velocity**	0.43	0.15	duration	0.15
mRNA Axon Bright	**Velocity**	0.75	0.58	duration	−0.02
mRNA Axon Dim	**Velocity**	0.52	0.21	duration	0.18
mRNA Dendrites Bright	**Velocity**	−0.34	0.11	duration	−0.001
mRNA Dendrites Dim	**Velocity**	0.54	0.22	duration	0.15

Bold indicates dominant variable.

## Discussion

This work exploited high-resolution imaging, image-processing, and analytical tools to generate a comprehensive quantitative assessment of mRNA transport in axons and dendrites of cultured hippocampal neurons at different stages of maturity. This quantitative approach enabled us to extend previous literature on mRNA transport through the identification and characterization of two distinct classes of non-mitochondrial mRNA, which appear to differ in mRNA content as well as transport characteristics. Our data also indicated interesting differences between mRNA transport in axons and dendrites, enabling us to propose intriguing hypotheses regarding varying roles for mRNA transport in neurites of different polarity and physiological function. These results provide a baseline for future work to uncover mechanisms involved in the coupling of mRNA transport to local translation.

Though the emphasis of this work was to provide new insights on mRNA transport, our concurrent analysis of mitochondria served multiple purposes. First, co-labeling mitochondria enabled us to distinguish between mitochondrial and non-mitochondrial mRNA, allowing greater clarity in interpreting previous studies that used general mRNA markers. Second, the fact that transport profiles of mRNA were distinct from those of mitochondria provided internal validation for the absence of global effects such as toxicity or changes in cellular geometry that would identically influence all cargoes. Third, we were able to confirm and extend previous quantitative analyses of mitochondrial transport in our cultured rat hippocampal model. Results and discussion pertinent to analysis of mitochondrial transport are presented as supplementary material (Fig. S7 and S8, Text S1).

### Transport profiles differ in bright versus dim populations of mRNA

Several research groups have studied the axonal and dendritic distributions of mRNA and local protein synthetic machinery [27], [28], [29]. However, details on the role of transport in achieving these distributions are less prevalent. Our quantitative characterization of mRNA transport builds on literature that has examined various aspects of neuronal mRNA transport, both qualitatively and quantitatively.

Brightly fluorescent particles of slowly moving mRNA exist in both axons and dendrites, and appear qualitatively similar to puncta identified as mRNA granules in previous studies on mRNA localization and transport [26], [30], [31]. Net axonal and dendritic velocities, measured within a 15 minute imaging window and extrapolated to longer time periods, are consistent with slow bulk axonal transport (0.1–1 mm/day), and in agreement with previous measurements of 0.5 mm/day reported in dendrites in radiolabel pulse-chase experiments [32]. Maximum velocities, both anterogradely and retrogadely, are slightly lower in axons (0.03–0.06 µm/sec), but agree exactly in dendrites (∼0.09–0.11 µm/sec) with the value of 0.1 µm/sec provided for motile mRNA granules in cortical neurites of unspecified polarity [26]. Minor differences in these values may result from developmental or physiological differences between our P1 neonatal hippocampal and published E18 cortical neurites. However, the likeliest source of discrepancy is the cutoff used to distinguish between stationary and motile puncta. With respect to the latter, our inclusion of particles with slow, but significant velocities at or above 0.001 µm/sec could have lowered the average rate of transport compared to values previously reported [26].

The observation of a weakly fluorescent pool of rapidly moving mRNA was novel and surprising. Net velocities for dim particles were over two orders of magnitude higher than for bright particles. However, the weaker fluorescence implies a smaller quantity of mRNA, offsetting this apparent increase in net transport. Differences in fluorescence intensity and transport parameters suggest different modes of packaging and regulation of transport as well as possible differences in function.

Because of the slow average velocities and our relatively low frame rate, it is not possible to infer the identity of motor proteins responsible for movement in either bright or dim pools of mRNA. However, cell biological and biochemical studies have identified RNA granule association with KIF5 (kinesin-1) and KIF3 (kinesin-2) in dendrites, KIF3C in axons, and kinesin-1 and dynein in Drosophila S2 cells [33], [34], [35], [36], [37]. A role for microtubule-dependent motor proteins in transporting mRNA is also consistent with reductions in mRNA localization within neurites following microtubule destabilization with colchicine [26], [38]. mRNA may also piggyback on ribosomes or other cargoes with which it has been reported to co-localize, including cytoskeletal elements such as actin [10], [35], [39].

Varying functional roles for bright and dim particles can also not be inferred from our analysis. However, based on the increased mobility of this dim pool, one intriguing hypothesis is that dim particles reflect a pool of specific transcripts quickly recruited in response to an unexpected stimulus, such as injury, the termination of axonal outgrowth, or synaptic activity. This hypothesis is consistent with the trafficking of Arc mRNA, which encodes a protein believed to be involved in the maintenance of LTP. Synaptic activity triggers the transport of Arc mRNA to activated synaptic sites [40], and it accumulates near stimulated synapses on a time course that coincides with the duration of protein synthesis during LTP [41]. Such a role would contrast with a more general role for maintenance of cellular infrastructure during growth and homeostasis, which could be reflected in the larger, slower granules. Such posited differences are conceptually similar to differences in mitochondrial populations, which are often stationary along the axon, but are more mobile when recruited to areas of high demand, such as an extending growth cone (Miller and Sheetz, 2006). An alternate hypothesis is that larger, brighter particles represent a multicomponent granular complex, while dim particles represent particles that are not yet incorporated into a substantial protein synthetic complex or smaller, more specialized translational entities. These hypotheses should be addressed in future studies that elucidate the identity of specific transcripts and components of the translational machinery that are transported in each pool.

### mRNA transport profiles differ in axons and dendrites

We identified several interesting differences in mRNA transport between axons and dendrites at varying stages of neurite maturity, in both bright and dim populations. Net velocities within a given neurite represent the combined influences of run velocities, net directionality, and the duration of movement of individual particles; thus, it was possible to identify the particular parameter or parameters responsible for any differences across experimental groups. For bright particles in axons, net velocities peaked at day 7 before falling again at day 12. This net positive effect stemmed primarily from increased velocity and an increased proportion of anterogradely moving particles on day 7 compared to days 4 and 12 (Fig. S5a and Fig. S6a). The subsequent reduction in net anterograde velocity at day 12 resulted from a slight decrease in anterograde velocity and an increase in stationary particles, at the expense of particles moving both anterogradely and retrogradely. These transport patterns were different from those of bright mRNA particles in dendrites, where net velocities were an order of magnitude smaller than in axons, and were retrograde at day 4 before changing directionality by day 12. This pattern was a result of a sharp transition from a predominantly retrograde pool of moving particles at day 4 to an anterograde pool by day 12 (Fig. 6b). In contrast to bright particles, dim particles had comparable net velocities in both axons and dendrites that were up to two orders of magnitude higher than those of bright particles in either type of neurite. Also in contrast to bright particles, the net velocities of dim particles increased dramatically between day 4 and 7 (Fig. 7c), coinciding with the timeline for synapse formation and stabilization (Fig. 1).

In comparing transport timelines in axons versus dendrites, the relative increase in net axonal mRNA velocity at an earlier time point compared to dendrites supports a model where local translation initially contributes to synapse stabilization *pre-synaptically*. Indeed, the observed increase in the net axonal velocity of bright and dim mRNA particles at day 7 coincides with the end stages of neurite outgrowth and the initial stages of synapse formation and stabilization (Fig. 1, [22], [23]). Such a timeline is also consistent with reported relationships between local translation and the effectiveness of neurotrophic signaling both during axonal outgrowth and pre-synaptic signaling [42], [43]. Most notably, protein synthesis in both the axon and dendrites is required for effective axonal guidance by brain-derived neurotrophic factor (BDNF) and neurotrophin-3 (NT-3) [15], [44]. Neurotrophins may also initiate early stages of synaptic strengthening, as axonally synthesized BDNF has been implicated in potentiating transmitter secretion from nearby synapses [15].

In contrast, net dendritic velocities of bright, slow mRNA particles illustrated a linear relationship between net velocities and the maturity of neurons, with the highest reported net velocities at day 12 coinciding with an increased duration of synaptic contact (Fig. 7). Increased and sustained dendritic mRNA transport at later time points is consistent with a role for local protein synthesis in the context of sustained synaptic connectivity for LTP, consolidation of long-term memory, and immunity versus long-term depression [9], [15], [16], [41], [45], [46], [47], [48], [49]. Several locally synthesized proteins relevant for such synaptic plasticity are candidates to be found in observed dendritic mRNA pools, including αCAMKII [50] and cytoplasmic polyadenylation element binding protein (CPEB), which upon increased translation, through its long-lasting transmissible conformation, serves to provide a “memory” of the synaptic stimulation [51].

### Conclusions

This work provides the first rigorous quantitative assessment of axonal and dendritic mRNA transport during central neuronal development. Significant differences in transport parameters for individual and pooled particles at different stages of neuronal maturity emphasize the dynamic nature of transport at multiple levels. As suggested for other cargoes, including mitochondria [18], [52], [53], [54], [55], [56], the dynamics of mRNA transport are likely to be driven by the functional demands of the cell. Future studies will uncover mechanisms initiating and regulating changes in mRNA transport as well as the identity of specific classes of transcripts that are subject to such regulation. Additionally, datasets of multiple mRNA and mitochondria transport parameters that we generated in this study will be essential for validating theoretical models of neuronal mRNA or mitochondrial transport. It is our belief that multi-disciplinary approaches spanning the computational and biological realms will yield tremendous progress in understanding pathways of local translation and its regulation in neuronal development, disease, and injury.

## Methods

### Ethics statement

All animal protocols were approved by University of Maryland Institutional Animal Care and Use Committee.

### Primary cell culture

Hippocampi were dissected from one-day-old Sprague-Dawley rats and maintained in ice-cold HBSS (2) media (HBSS 500 mL, D glucose .4 g, HEPES .834 g, Penicillin 15 mL). They were then incubated with 0.05% DNase (1.4 M MgSO4 , HBSS 100%) and Mixture A (PBS 100%, DL-Cysteine HCL 1.6 mM, BSA 3.7 µM, D-glucose 34.6 mM, Papin 21 µM) for 30 minutes at 37°C under agitation at 100 rpm. Following trituration, cells were pelleted at 234× g for 3 minutes before resuspension in growth media (Neurobasal media supplemented with 2% B-27). Finally, cells were plated on coverslips coated with 1 mg/ml polylysine at a density of 20,000 cells in 500 µL. All cells were maintained and imaged at 37°C and 5% Co2.

### Immunofluorescence

Hippocampal cells were fixed with 4% paraformaldehyde in PBS for 10 minutes and rinsed with PBS three times. Following permeabilization with 0.2% Triton X-100 in PBS, the cells were blocked with 10% Fetal goat serum and 3% BSA for 30 minutes. A 1∶1000 dilution of SMI-31 (Abcam Inc., Cambridge, MA), 1∶500 dilution of MAP2 (Abcam Inc., Cambridge, MA), or 1∶200 dilution of Synapsin I (Sigma-Aldrich Corp., St. Louis, MO) in BSA was applied for an hour at room temperature, followed by three washes in PBS. Fluorescently labeled secondary antibody was applied subsequently for 1 hr at 37°C, followed again by three washes in PBS. Finally, coverslips were mounted on a slide in the presence of Vectashield (Vector Laboratories, Inc., Burlingame, CA). For co-labeling with RNA, cells were incubated with 500 nM of Syto after application of the secondary antibody.

### mRNA and mitochondrial labeling

For mRNA labeling, 500 nM solution of RNASelect green fluorescent cell stain (Syto, Excitation 490 nm, Emission 530 nm) in cell media was prepared. This solution was pre-warmed at 37°C prior to application and used immediately. The cells were incubated with 500 µL of the 500 nM labeling solution for 20 minutes at 37°C. After this, cells were rinsed once with cell-culture medium. Mitochondria (Excitation 579 nm, Emission 599 nm) were labeled by incubating dissociated neurons in a 1∶10,000 dilution of mitochondrial dye, MitoTracker Red for 10 minutes at 37°C, followed by rinsing with the cell-culture media. The neurons were labeled on days 4,7, and 12. The imaging was performed immediately after application of the probes.

### Fluorescence Microscopy

Imaging was performed on an inverted TE-2000E microscope (Nikon, Melville, NY) outfitted with a Lumen-PRO2000 (Prior Scientific, Rockland, MA) illumination system and Chroma filters (Bellows Falls, VT. EPI: 488 nm, Emission 530 nm). Additionally, a custom built chamber (Precision Plastics, Beltsville, MD) maintained temperature, humidity, and CO2 levels during imaging. DIC and Fluorescence images were captured for 15 minutes every 15 seconds using a 40× objective with 200 ms exposure time. DIC, Syto RNA (490 nm excitation), and Mitotracker (570 nm excitation) channels were captured at the same time point, sequentially within 1–2 seconds, accounting for filter changes.

### RNase Treatment

After incubation with Syto, imaging and live imaging was performed as mentioned. Then, cells were fixed with 4% paraformaldehyde for 10 min. After fixation, cells were permeabilized with 0.5% Triton X-100 for 5 min and incubated with RNase A 10 µg/ml in Tris-buffered solution and for 1 hr at 37°C. A second image was taken of the labeled cell, and the intensities of the two signals were compared.

### Image Analysis

Image analysis was performed either on MATLAB (MathWorks, Natick, MA) or ImageJ (NIH). To analyze the movement of mitochondria and mRNA particles in the axon over time, a custom program was used to create kymographs from time lapse movies as previous described [57]. Trajectories of mRNA particles that did not overlap with those of MitoTracker were concluded to be mRNA particles. The likelihood of a false positive (i.e., a mitochondrial particle that was mistakenly identified as a non-mitochondrial particle) was very low, owing to the considerably brighter fluorescence intensity of MitoTracker compared to Syto. mRNA particles from non-mitochondrial mRNA particles were analyzed separately. A series of image processing steps was performed to confirm and better visualize dim mRNA particles (Fig. S3). First, the kymograph of interest was processed by iteratively overlaying 300 images of the same kymograph, which were made 50% transparent (Gnu Image Manipulation Program, http://www.gimp.org). Each overlay resulted in a slight increase in signal to noise ratio, allowing enhancement of dim signals. The resulting kymograph was then contrast enhanced or inverted to visualize the dim mRNA moving particle (ImageJ). Finally, local contrast enhancement was performed on each particle. Contrast enhancement with a similar mask was performed on both regions of interest (ROI) and non-ROI regions, to ascertain that local contrast enhancement did not result in a false positive particle.

### Data Analysis

For directionality analyses, the following sample sizes (numbers of neurons) for each category were used; bright mRNA particles in axons: day 4: N = 11, day 7: N = 10, day 12: N = 11; bright mRNA particles in dendrites: day 4: N = 4, day 7: N = 5, day 12: N = 6; dim mRNA particles in axons: day 4: N = 5, day 7: N = 8, day 12: N = 11; dim mRNA particles in dendrites: day 4: N = 3, day 7: N = 5, day 12: N = 6; mitochondria in axons: day 4: N = 11, day 7: N = 10, day 12: N = 11; and mitochondria in dendrites: day 4: N = 4, day 7 N = 4, day 12: N = 6.

In addition, for net velocity, velocity, and distribution analyses, following sample sizes (number of particles) for each category were used: bright mRNA particles in axons: day 4: N = 34, day 7: N = 53, day 12: N = 52; bright mRNA particles in dendrites: day 4: N = 11, day 7: N = 14, day 7: N = 27; dim mRNA particles in axons: day 4: N = 31, day 7: N = 66, day 12: N = 77; dim mRNA particles in dendrites: day 4: N = 25, day 7: N = 56, day 12: N = 39; mitochondria in axons: day 4: N = 69, day 7: N = 82, day 12: N = 88; mitochondria in dendrites: day 4: N = 35, day 7: N = 42, day 12: N = 52.

### Maximum and average velocity

Particles with an average velocity of greater than 0.001 µm/sec in either direction (0.1 mm/day) were classified as moving, based on minimum velocities measured in a previous study [32]. Moving particles were segregated and further analyzed. The maximum velocity in each direction for each particle was calculated. In addition, the average instantaneous velocity in each direction for particles was calculated.

### Directionality Analysis

Particles not scored as moving, as defined above, were considered stationary. Most moving particles spent the majority of their time (>80%) moving unidirectionally; however, a small fraction of particles changed directions multiple times during a trajectory. These wiggling particles were classified as moving bidirectionally if their net displacement was less than 0.001 µm.

### Duration of directional movement

Only particles considered moving, as defined above, were analyzed for this parameter. The cumulative duration that each particle moved during its entire trajectory was calculated for both the anterograde and retrograde direction.

### Net Velocity

The net velocity for each particle was calculated using the equation below.
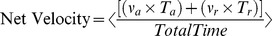
where ν_a_ = average anterograde velocity, ν_r_ = average retrograde velocity, T_a_ = Time spent in the anterograde direction, T_r_ = Time spent in the retrograde direction. Graphs of the net velocities were obtained by averaging all particles from all neurites.

### Statistics

Means were compared using ANOVA followed by Tukey's post hoc analysis. Distributions were compared using Kolmogrov-Smirnov Test. We have also performed multiple regression, with net velocity as the dependent variable and velocity and duration as independent variables. All statistical analysis was performed using SAS software (Cary, NC).

## Supporting Information

Figure S1 **Hippocampal neurons were co-labeled with Syto nucleic acid stain and Hoechst nuclear stain.** Syto and Hoechst co-localize within the cell body (arrows), but there is no Hoechst labeling in neurites, which display Syto fluorescence. (A) Neurons stained with Syto nucleic acid stain (green). (B) Neurons stained with Hoechst nuclear stain (blue). (C) Double-label of Syto (green) and Hoechst (blue). Bar is 20 µm.
(TIFF)

Figure S2 **RNase A treatment was done to ascertain that dim particles are in fact mRNA particles.** (A) Kymographs of mRNA particles pre-RNase treatment. (B) Neurons stained with Syto nucleic acid stain (green). (C) Neurons stained with Syto nucleic acid stain frame #3 (green). (D) Neurons stained with Syto nucleic acid stain frame #11 (green). The arrows indicate corresponding “dim” particle as it moves over five minutes. The bright particle indicated with down arrow had an average intensity of 9.26 arbitrary units, and dim particle indicted with up arrow had an average intensity of 0.98 arbitrary units. Values account for background subtraction. (E) Corresponding neuron after RNase treatment showing no Syto signaling in the neurites. Puncta indicated by arrows pre-RNase had intensities indistinguishable from background levels. All images are shown contrast enhanced, confirming full suppression of neurite fluorescence. (F) Cropped kymograph enlarged, from (A). (G) Corresponding region from (B) enlarged, including dim particle. Bar is 20 µm.
(TIFF)

Figure S3 **Methods of image analysis.** Time-lapse images of neurites were taken under DIC and fluorescence conditions. After classifying the neurite as an axon or dendrite using the DIC image, RNA particles (Syto, green) and mitochondrial particles (Mitotracker, red) were fluorescently labeled to identify respective particles. Contrast enhancement and several controls were performed to confirm dim particles. (A) Non-mitochondrial mRNA kymograph without contrast enhancement. (B) ) Kymograph from (A) following iterative overlay of 50% transparent image to visualize dim moving particles (C) Kymograph from (B) inverted using ImageJ to visualize dim particles (arrow). (D) Kymograph from (B). (E) Kymograph from (D) following contrast enhancement in ImageJ to visualize dim particles (arrow). (F) Kymograph from (D) inverted and contrast enhanced (arrow). (G) Kymograph from (B). Local contrast enhancement improved dim particle visualization. (H) Selection of background region with no apparent particles (yellow lines) for contrast enhancement. (I) Kymograph from (H) following local contrast enhancement does not indicate a particle trajectory in selected region (oval), confirming validity of contrast enhancement. (J) Kymograph from (B). (K) Selection of region of interest (ROI-yellow lines) for local contrast enhancement to visualize dim particles. (L) Kymograph from (K) following local contrast enhancement to visualize dim particles (arrow).
(TIFF)

Figure S4 **Time-lapse images of neurites were taken under DIC and fluorescence conditions.** After classifying the neurite as an axon or dendrite using the DIC image, RNA particles (Syto, green) and mitochondrial particles (Mitotracker, red) were fluorescently labeled to identify respective particles. (A) Whole kymograph following iterative overlay of 50% transparent image to visualize dim moving particles (arrows). (B) Kymograph from (A) cropped upper region to visualize dim particles (arrows). (C) Kymograph from (B) inverted to visualize dim particles (arrows). (D) Kymograph from (A) after cropping lower region to visualize dim particles (arrows). (E) Kymograph from (C) inverted to visualize dim particles (arrows). Bar is 20 µm.
(TIFF)

Figure S5 **Maximum track velocity of mRNA in axons and dendrites.** Particles were considered moving if their average velocity in either direction was greater than 0.001 µm/sec (0.1 mm/day). (A) Maximum track velocity of bright and dim mRNAs moving through axons in the anterograde direction. Within the bright mRNA population, particles moved more slowly at day 12 compared with day 7 (p<0.002, K-S test). (B) Maximum track velocity of bright and dim mRNA particles moving through axons in the retrograde direction. Within the bright mRNA population, there is a rightward shift. Overall, more particles moved faster at day 7 compared to day 4 (p<0.03, K-S test). (C) Maximum track velocity of bright and dim mRNA particles moving along dendrites in the anterograde direction. Within the bright mRNA population, there was a leftward shift. Significantly different velocities were observed for all days; however, at day 12 there were more particles that moved slowly (p<0.05, K-S test). Within the dim population, there were significantly more particles that moved faster at day 12 compared to day 7 (p<0.01, K-S test). (D) Maximum track velocity of bright and dim mRNA particles moving through dendrites in the retrograde direction.
(TIFF)

Figure S6 **Distributions of track movement durations of mRNA in axons and dendrites were calculated from kymographs for days 4, 7, and 12.** Only particles classified as moving were analyzed. Individual particle durations are presented as cumulative histograms. (A) Track durations of bright mRNA particles moving through axons in the anterograde direction. (B) Track durations of bright mRNA particles moving through axons in the retrograde direction. (C) Track durations of bright mRNA particles moving through dendrites in the anterograde direction. (D) Track durations of bright mRNA particles moving through dendrites in the retrograde direction. (E) Track durations of dim mRNA particles moving through axons in the anterograde direction. (F) Track durations of dim mRNA particles moving through axons in the retrograde direction. Distributions were significantly different for days 4 vs. day 12 (p<0.03, K-S test) and at day 7 vs. day 12 (p<0.04, K-S test). (G) Track durations of dim mRNA particles moving along dendrites in the anterograde direction. (H) Track durations of dim mRNAs particles moving through dendrites in the retrograde direction.
(TIFF)

Figure S7 **Maximum track velocities and movement durations of mitochondria in axons and dendrites were calculated from kymograph for days 4, 7, and 12, and are presented as cumulative histograms.** (A) Maximum track velocity of mitochondria moving through axons in the anterograde direction. Distributions of maximum mitochondrial velocities were significantly different at day 4 compared to day 12 (p<0.005, , K-S test), and at day 7 compared to day 12 (p<0.001, K-S test). (B) Maximum track velocities of mitochondria moving through axons in the retrograde direction. Distributions of maximum mitochondrial velocities were significantly different at day 4 compared to day 7 (p<0.03, K-S test), and at day 7 compared to day 12 (p<0.03, K-S test). (C) Maximum track velocitis of mitochondria moving through dendrites in the anterograde direction. Distributions of maximum mitochondrial velocities were significantly different at day 4 compared to day7 (p<0.02, K-S test) at day 4 compared to day 12 (p<0.001, K-S test) and at day 7 compared to day 12 (p<0.008, K-S test). (D) Maximum track velocity of mitochondria moving through axons in the retrograde direction. Distributions of maximum mitochondrial velocities were significantly different at day 4 compared day 12 (p<0.02, K-S test). (E) Track durations of mitochondria moving through axons in the anterograde direction. Distributions of durations were significantly different at day 4 compared to day 12 (p<0.0007, K-S test), and at day 7 compared to day 12 (p<0.0001, K-S test). (F) Track durations of mitochondria moving through axons in the retrograde direction. Distributions of durations were significantly different at day 4 compared to day 12 (p<0.0001, K-S test) and at day 7 compared to day 12 (p<0.001, K-S test). (G) Track durations of mitochondria moving through dendrites in the anterograde direction. (H) Track durations of mitochondria moving through dendrites in the retrograde direction. Distributions of durations were significantly different at day 4 compared to day 12 (p<0.02, K-S test).
(TIFF)

Figure S8 **Net directionality of mitochondria particle movement over its lifetime.** Particles were considered moving if their average velocity in either direction was greater than 0.001 µm/sec (0.1 mm/day). Particles that did not meet this criterion were designated stationary. (A) Percent of mitochondria in axons in each state for days 4, 7, and 12. There are significantly more particles moving in anterograde direction at day 7 compared to day 4 *p<0.05 (ANOVA: Tukey). (B) Percent of mitochondria in dendrites in each state for days 4, 7, and 12. Average net velocity of individual mitochondria particles in axons and dendrites. (C) Average of net mitochondrial velocity moving through axons (D) Average of net mitochondrial velocity moving through dendrites. The net velocity is higher at day 7 compared to day 4 (p<0.05) and at day 12 vs. day 4 (p<0.05; ANOVA:Tukey). Plotted values indicate mean ± SEM.
(TIFF)

Table S1 **Summary of maximum velocities for various classes of labeled cargoes.** * Significant difference (day 4 vs. day 12 *p<0.05). ⋆ Significant difference (day 4 vs. day 7 ⋆p<0.05). ✶ Significant difference (day 7 vs. day 12 ✶p<0.05).
(DOC)

Table S2 **Summary of average velocities for various classes of labeled cargoes.** * Significant difference (day 4 vs. day 12 *p<0.05). ⋆ Significant difference (day 4 vs. day 7 ⋆p<0.05). ✶ Significant difference (day 7 vs. day 12 ✶p<0.05).
(DOC)

Table S3 **Summary of duration spent moving in each direction for various classes of labeled cargoes.** * Significant difference (day 4 vs. day 12 *p<0.05). ⋆ Significant difference (day 4 vs. day 7 ⋆p<0.05). ✶ Significant difference (day 7 vs. day 12 ✶p<0.05).
(DOC)

Table S4 **Summary of net velocities for various classes of labeled cargoes.** * Significant difference (day 4 vs. day 12 *p<0.05). ✶ Significant difference (day 4 vs. day 7 ✶p<0.05).
(DOC)

Text S1 **Results related to Mitochondria transport analysis are presented in this section.**
(DOC)
