# Supplementary figures and images for: A Comparative Quantitative Assessment of Axonal and Dendritic mRNA Transport in Maturing Hippocampal Neurons
Source: PLoS One. 2013 Jul 22;8(7):e65917. doi: 10.1371/journal.pone.0065917 (PMC3718819; doi:10.1371/journal.pone.0065917)

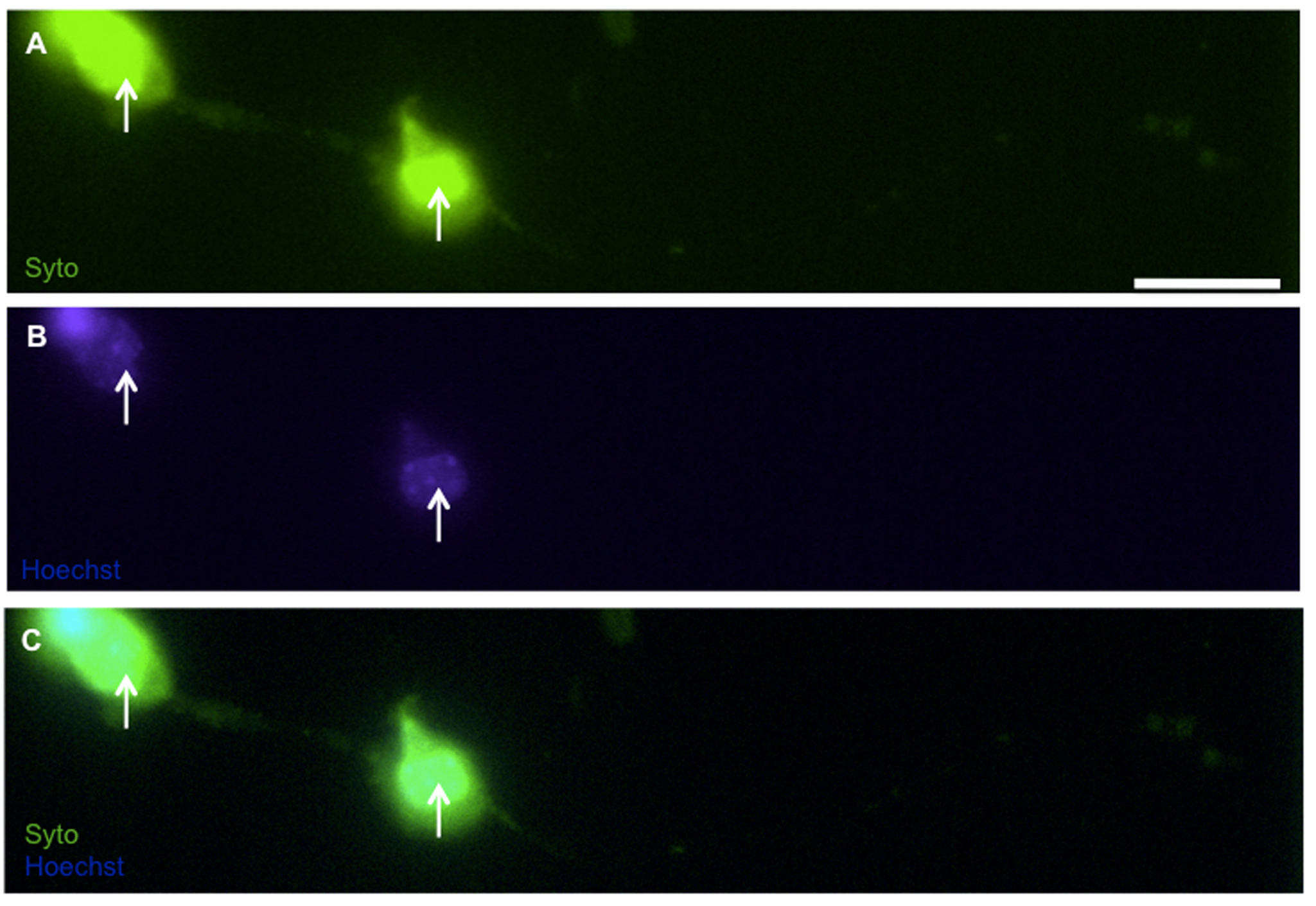

Supplement: Figure S1 — Hippocampal neurons were co-labeled with Syto nucleic acid stain and Hoechst nuclear stain. Syto and Hoechst co-localize within the cell body (arrows), but there is no Hoechst labeling in neurites, which display Syto fluorescence. (A) Neurons stained with Syto nucleic acid stain (green). (B) Neurons stained with Hoechst nuclear stain (blue). (C) Double-label of Syto (green) and Hoechst (blue). Bar is 20 µm. (TIFF) [file pone.0065917.s001.tif]

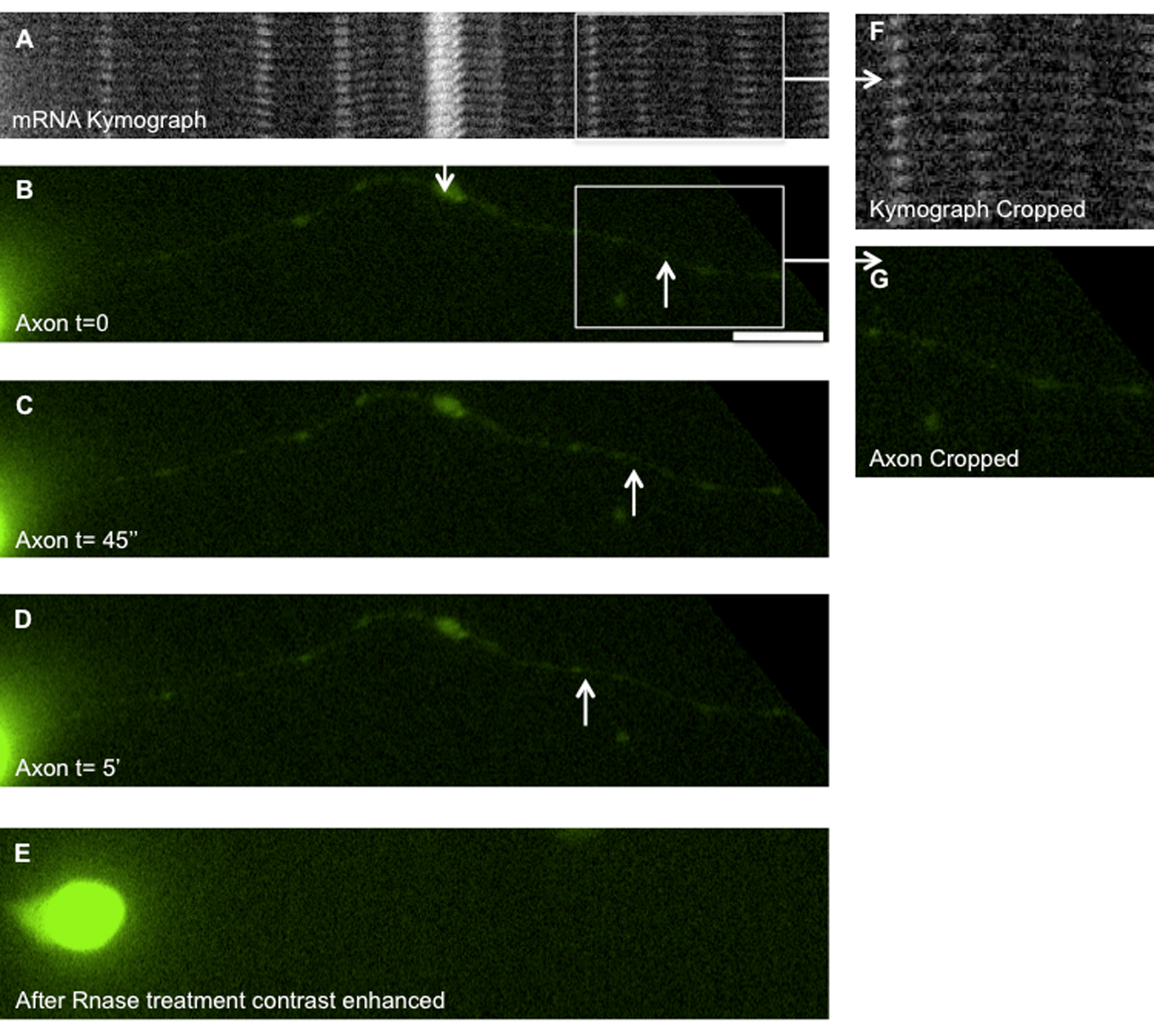

Supplement: Figure S2 — RNase A treatment was done to ascertain that dim particles are in fact mRNA particles. (A) Kymographs of mRNA particles pre-RNase treatment. (B) Neurons stained with Syto nucleic acid stain (green). (C) Neurons stained with Syto nucleic acid stain frame #3 (green). (D) Neurons stained with Syto nucleic acid stain frame #11 (green). The arrows indicate corresponding “dim” particle as it moves over five minutes. The bright particle indicated with down arrow had an average intensity of 9.26 arbitrary units, and dim particle indicted with up arrow had an average intensity of 0.98 arbitrary units. Values account for background subtraction. (E) Corresponding neuron after RNase treatment showing no Syto signaling in the neurites. Puncta indicated by arrows pre-RNase had intensities indistinguishable from background levels. All images are shown contrast enhanced, confirming full suppression of neurite fluorescence. (F) Cropped kymograph enlarged, from (A). (G) Corresponding region from (B) enlarged, including dim particle. Bar is 20 µm. (TIFF) [file pone.0065917.s002.tif]

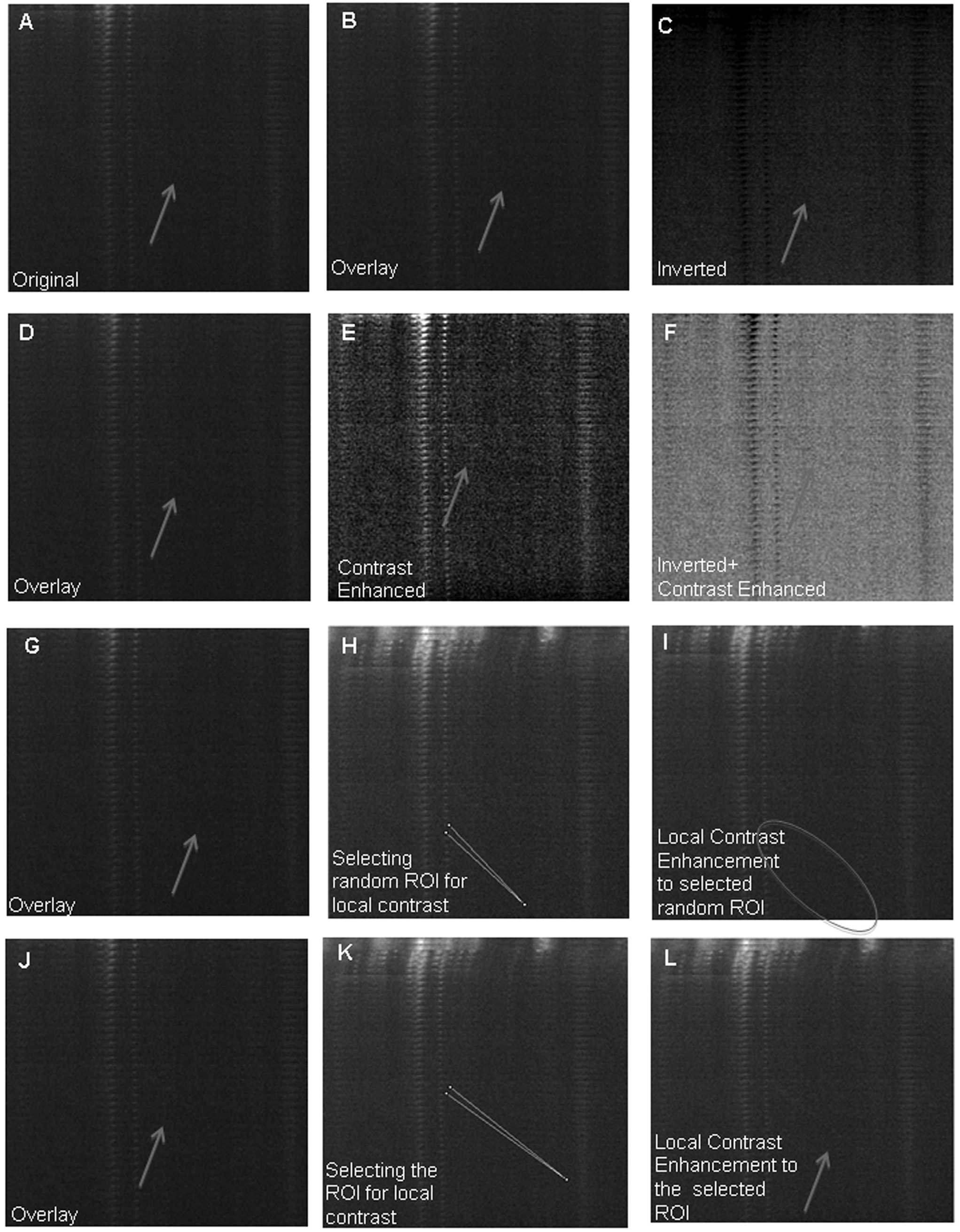

Supplement: Figure S3 — Methods of image analysis. Time-lapse images of neurites were taken under DIC and fluorescence conditions. After classifying the neurite as an axon or dendrite using the DIC image, RNA particles (Syto, green) and mitochondrial particles (Mitotracker, red) were fluorescently labeled to identify respective particles. Contrast enhancement and several controls were performed to confirm dim particles. (A) Non-mitochondrial mRNA kymograph without contrast enhancement. (B) ) Kymograph from (A) following iterative overlay of 50% transparent image to visualize dim moving particles (C) Kymograph from (B) inverted using ImageJ to visualize dim particles (arrow). (D) Kymograph from (B). (E) Kymograph from (D) following contrast enhancement in ImageJ to visualize dim particles (arrow). (F) Kymograph from (D) inverted and contrast enhanced (arrow). (G) Kymograph from (B). Local contrast enhancement improved dim particle visualization. (H) Selection of background region with no apparent particles (yellow lines) for contrast enhancement. (I) Kymograph from (H) following local contrast enhancement does not indicate a particle trajectory in selected region (oval), confirming validity of contrast enhancement. (J) Kymograph from (B). (K) Selection of region of interest (ROI-yellow lines) for local contrast enhancement to visualize dim particles. (L) Kymograph from (K) following local contrast enhancement to visualize dim particles (arrow). (TIFF) [file pone.0065917.s003.tif]

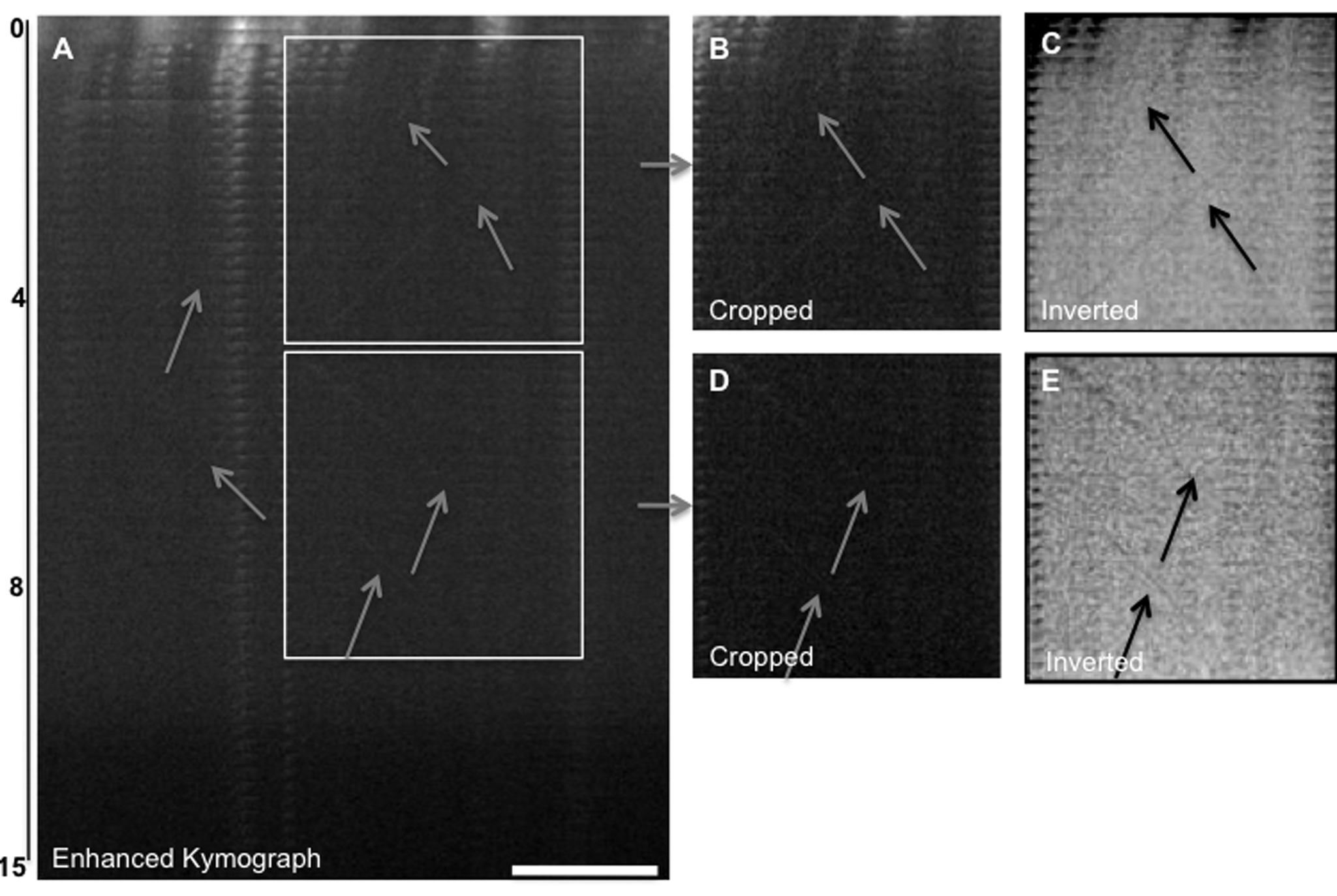

Supplement: Figure S4 — Time-lapse images of neurites were taken under DIC and fluorescence conditions. After classifying the neurite as an axon or dendrite using the DIC image, RNA particles (Syto, green) and mitochondrial particles (Mitotracker, red) were fluorescently labeled to identify respective particles. (A) Whole kymograph following iterative overlay of 50% transparent image to visualize dim moving particles (arrows). (B) Kymograph from (A) cropped upper region to visualize dim particles (arrows). (C) Kymograph from (B) inverted to visualize dim particles (arrows). (D) Kymograph from (A) after cropping lower region to visualize dim particles (arrows). (E) Kymograph from (C) inverted to visualize dim particles (arrows). Bar is 20 µm. (TIFF) [file pone.0065917.s004.tif]

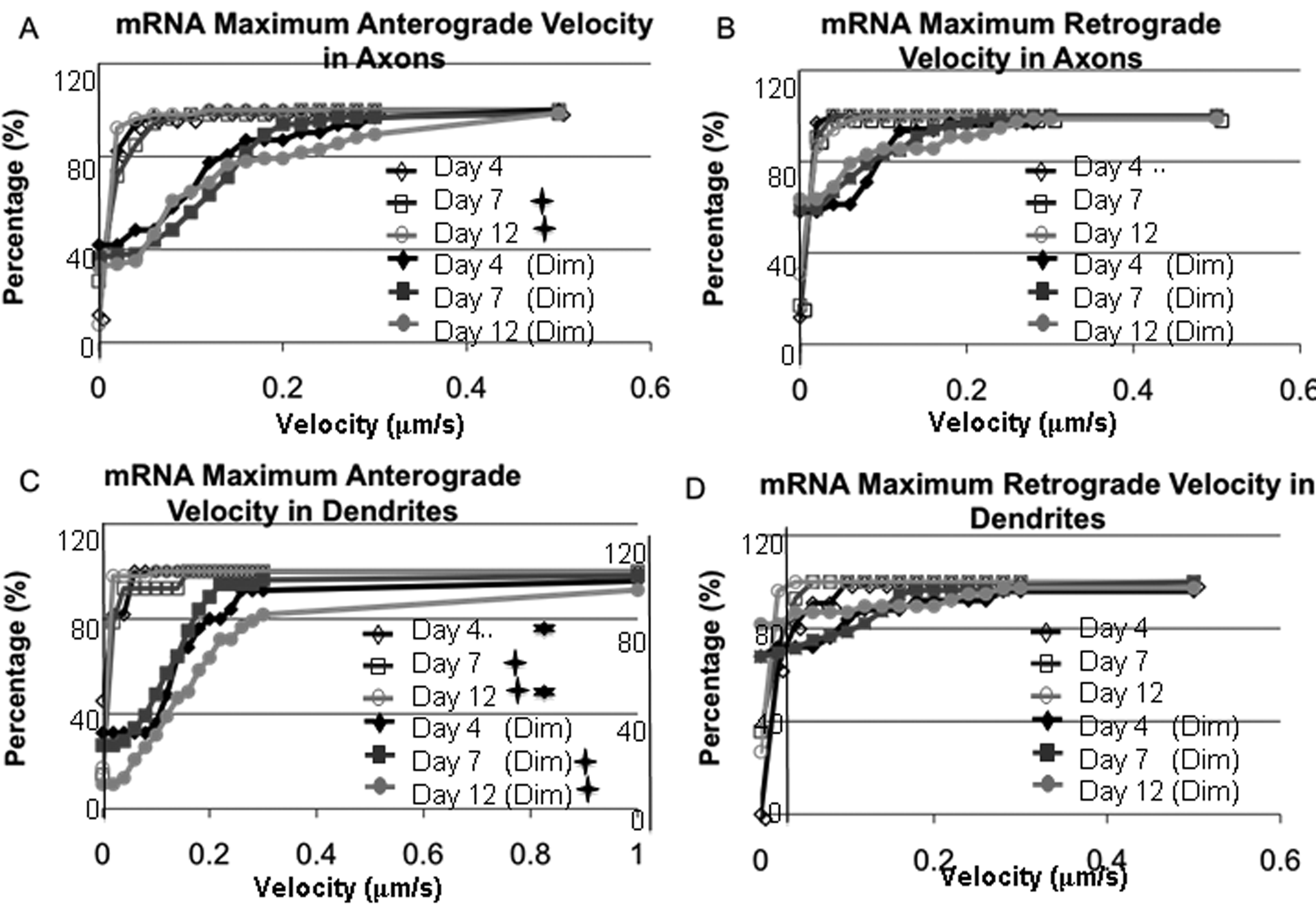

Supplement: Figure S5 — Maximum track velocity of mRNA in axons and dendrites. Particles were considered moving if their average velocity in either direction was greater than 0.001 µm/sec (0.1 mm/day). (A) Maximum track velocity of bright and dim mRNAs moving through axons in the anterograde direction. Within the bright mRNA population, particles moved more slowly at day 12 compared with day 7 (p<0.002, K-S test). (B) Maximum track velocity of bright and dim mRNA particles moving through axons in the retrograde direction. Within the bright mRNA population, there is a rightward shift. Overall, more particles moved faster at day 7 compared to day 4 (p<0.03, K-S test). (C) Maximum track velocity of bright and dim mRNA particles moving along dendrites in the anterograde direction. Within the bright mRNA population, there was a leftward shift. Significantly different velocities were observed for all days; however, at day 12 there were more particles that moved slowly (p<0.05, K-S test). Within the dim population, there were significantly more particles that moved faster at day 12 compared to day 7 (p<0.01, K-S test). (D) Maximum track velocity of bright and dim mRNA particles moving through dendrites in the retrograde direction. (TIFF) [file pone.0065917.s005.tif]

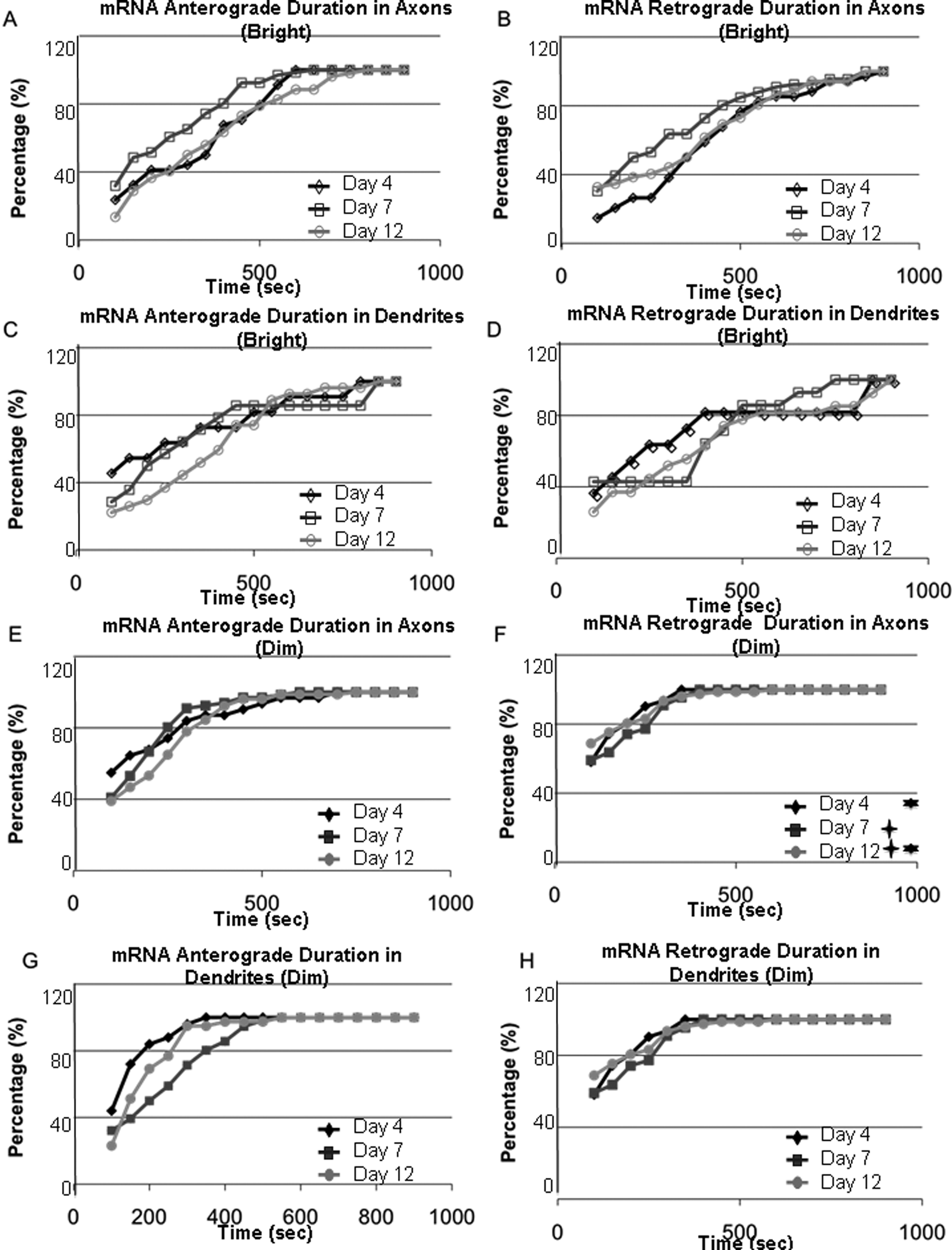

Supplement: Figure S6 — Distributions of track movement durations of mRNA in axons and dendrites were calculated from kymographs for days 4, 7, and 12. Only particles classified as moving were analyzed. Individual particle durations are presented as cumulative histograms. (A) Track durations of bright mRNA particles moving through axons in the anterograde direction. (B) Track durations of bright mRNA particles moving through axons in the retrograde direction. (C) Track durations of bright mRNA particles moving through dendrites in the anterograde direction. (D) Track durations of bright mRNA particles moving through dendrites in the retrograde direction. (E) Track durations of dim mRNA particles moving through axons in the anterograde direction. (F) Track durations of dim mRNA particles moving through axons in the retrograde direction. Distributions were significantly different for days 4 vs. day 12 (p<0.03, K-S test) and at day 7 vs. day 12 (p<0.04, K-S test). (G) Track durations of dim mRNA particles moving along dendrites in the anterograde direction. (H) Track durations of dim mRNAs particles moving through dendrites in the retrograde direction. (TIFF) [file pone.0065917.s006.tif]

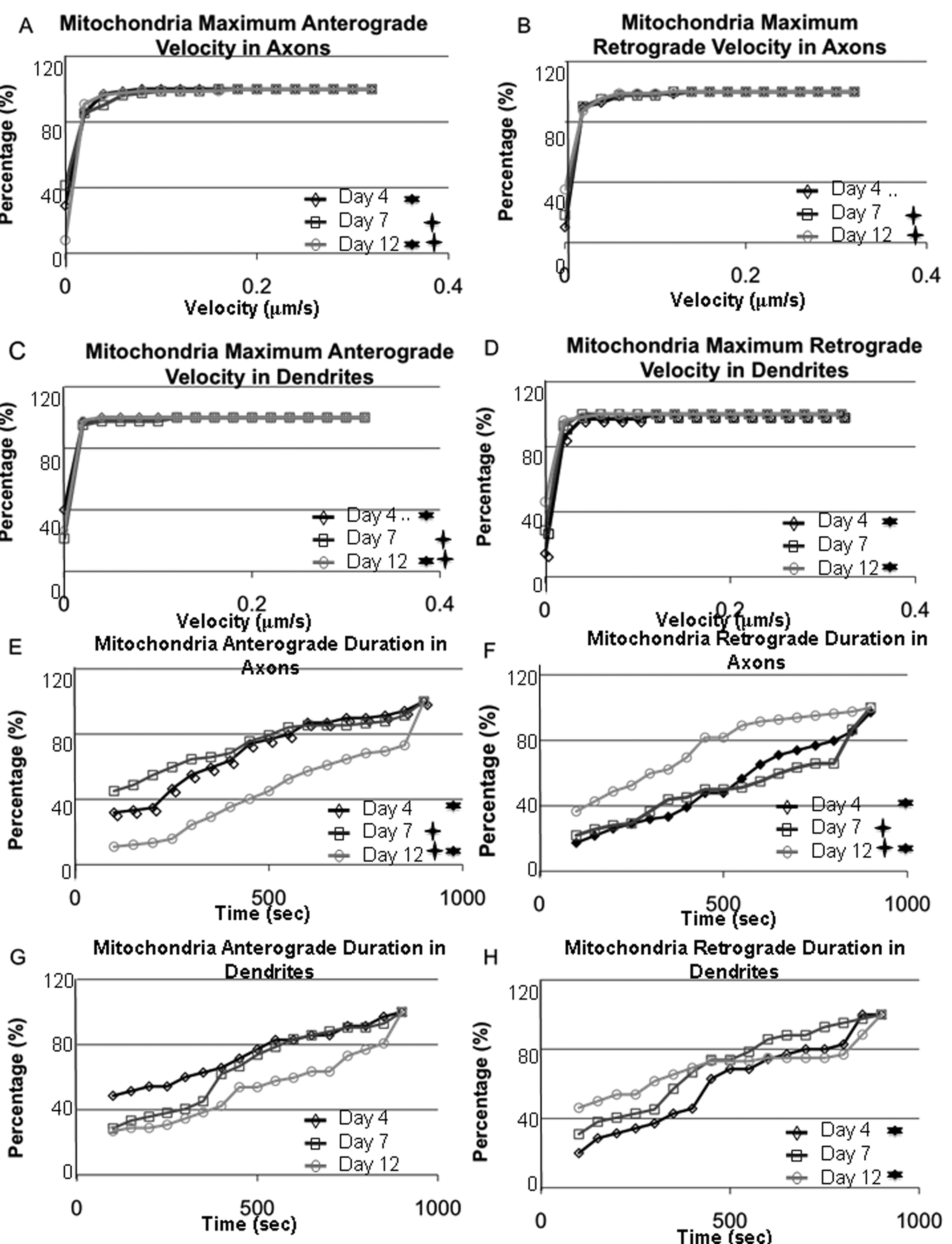

Supplement: Figure S7 — Maximum track velocities and movement durations of mitochondria in axons and dendrites were calculated from kymograph for days 4, 7, and 12, and are presented as cumulative histograms. (A) Maximum track velocity of mitochondria moving through axons in the anterograde direction. Distributions of maximum mitochondrial velocities were significantly different at day 4 compared to day 12 (p<0.005, , K-S test), and at day 7 compared to day 12 (p<0.001, K-S test). (B) Maximum track velocities of mitochondria moving through axons in the retrograde direction. Distributions of maximum mitochondrial velocities were significantly different at day 4 compared to day 7 (p<0.03, K-S test), and at day 7 compared to day 12 (p<0.03, K-S test). (C) Maximum track velocitis of mitochondria moving through dendrites in the anterograde direction. Distributions of maximum mitochondrial velocities were significantly different at day 4 compared to day7 (p<0.02, K-S test) at day 4 compared to day 12 (p<0.001, K-S test) and at day 7 compared to day 12 (p<0.008, K-S test). (D) Maximum track velocity of mitochondria moving through axons in the retrograde direction. Distributions of maximum mitochondrial velocities were significantly different at day 4 compared day 12 (p<0.02, K-S test). (E) Track durations of mitochondria moving through axons in the anterograde direction. Distributions of durations were significantly different at day 4 compared to day 12 (p<0.0007, K-S test), and at day 7 compared to day 12 (p<0.0001, K-S test). (F) Track durations of mitochondria moving through axons in the retrograde direction. Distributions of durations were significantly different at day 4 compared to day 12 (p<0.0001, K-S test) and at day 7 compared to day 12 (p<0.001, K-S test). (G) Track durations of mitochondria moving through dendrites in the anterograde direction. (H) Track durations of mitochondria moving through dendrites in the retrograde direction. Distributions of durations were significantly differ [file pone.0065917.s007.tif]

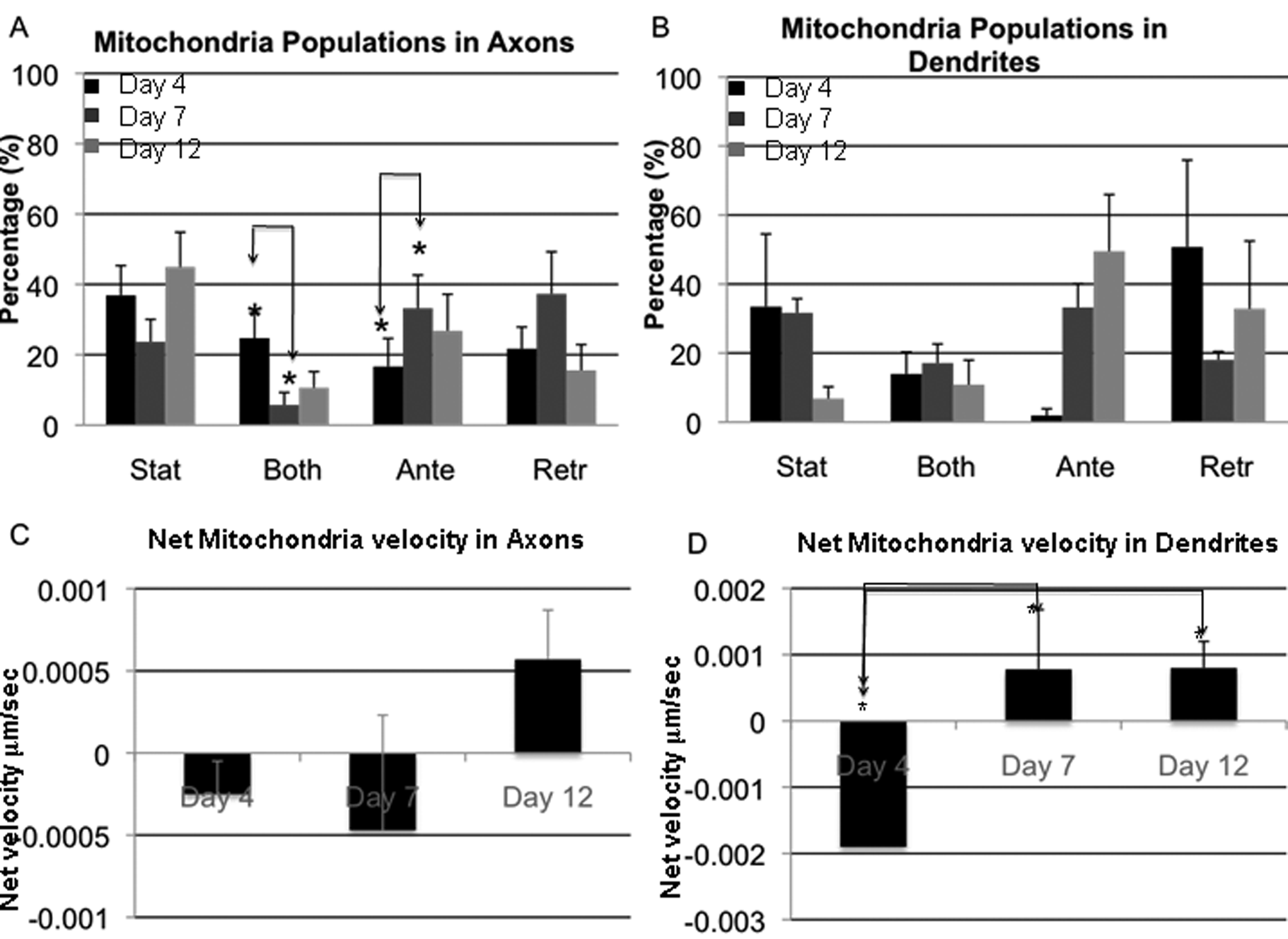

Supplement: Figure S8 — Net directionality of mitochondria particle movement over its lifetime. Particles were considered moving if their average velocity in either direction was greater than 0.001 µm/sec (0.1 mm/day). Particles that did not meet this criterion were designated stationary. (A) Percent of mitochondria in axons in each state for days 4, 7, and 12. There are significantly more particles moving in anterograde direction at day 7 compared to day 4 *p<0.05 (ANOVA: Tukey). (B) Percent of mitochondria in dendrites in each state for days 4, 7, and 12. Average net velocity of individual mitochondria particles in axons and dendrites. (C) Average of net mitochondrial velocity moving through axons (D) Average of net mitochondrial velocity moving through dendrites. The net velocity is higher at day 7 compared to day 4 (p<0.05) and at day 12 vs. day 4 (p<0.05; ANOVA:Tukey). Plotted values indicate mean ± SEM. (TIFF) [file pone.0065917.s008.tif]
