# Supplementary material for: A Comparative Quantitative Assessment of Axonal and Dendritic mRNA Transport in Maturing Hippocampal Neurons
Source: PLoS One. 2013 Jul 22;8(7):e65917. doi: 10.1371/journal.pone.0065917 (PMC3718819; doi:10.1371/journal.pone.0065917)
Supplement: Table S1 — Summary of maximum velocities for various classes of labeled cargoes. * Significant difference (day 4 vs. day 12 *p<0.05). ⋆ Significant difference (day 4 vs. day 7 ⋆p<0.05). ✶ Significant difference (day 7 vs. day 12 ✶p<0.05). (DOC) [file pone.0065917.s009.doc]

Table S1: Summary of maximum velocities for various classes of labeled cargoes. * Significant difference (day 4 vs. day 12 *p<.05).  Significant difference (p<.05). Significant difference (day 7 vs. day 12 p<.05).

| **Average Maximum Velocity** | **Day 4** | **Day 7** | **Day 12** |
| --- | --- | --- | --- |
| mRNA Axon anterograde (Dim) | 0.08± .02 | 0.09±.01 | 0.11±.01 |
| mRNA Axon retrograde (Dim) | -0.04±.01 | -0.04±.007 | -0.05±.01 |
| mRNA Dendrite anterograde (Dim) | 0.13±.03 | 0.12±.02 | 0.2±.03 |
| mRNA Dendrite retrograde (Dim) | -0.05±.02 | -0.04±.009 | -0.07±.05 |
| mRNA Axon anterograde (Bright) | 0.01±.003 | 0.02±.004 | 0.007±.003 |
| mRNA Axon retrograde (Bright) | -0.004±.0009 | -0.006±.001 | -0.008±.002 |
| mRNA Dendrite anterograde (Bright) | 0.008±.004 | 0.02±.005 | 0.008±.003 |
| mRNA Dendrite retrograde (Bright) | -0.02±.009[*] | -0.01±.003 | -0.005±.001[*] |
| Mitochondria Axon anterograde | 0.008±.001 | 0.01±.002 | 0.008±.002 |
| Mitochondria Axon retrograde | -0.009±.002 | -0.008±.002 | -0.007±.001 |
| Mitochondria Dendrite anterograde | 0.002±.0008 | 0.007±.003 | 0.004±.0007 |
| Mitochondria Dendrite retrograde | -0.009±.003[*] | -0.004±.001 | -0.003±.001[*] |
