# Supplementary material for: A Comparative Quantitative Assessment of Axonal and Dendritic mRNA Transport in Maturing Hippocampal Neurons
Source: PLoS One. 2013 Jul 22;8(7):e65917. doi: 10.1371/journal.pone.0065917 (PMC3718819; doi:10.1371/journal.pone.0065917)
Supplement: Table S2 — Summary of average velocities for various classes of labeled cargoes. * Significant difference (day 4 vs. day 12 *p<0.05). ⋆ Significant difference (day 4 vs. day 7 ⋆p<0.05). ✶ Significant difference (day 7 vs. day 12 ✶p<0.05). (DOC) [file pone.0065917.s010.doc]

Table S2: Summary of average velocities for various classes of labeled cargoes. * Significant difference (day 4 vs. day 12 p<.05). Significant difference (day vs. day 7 p< .05). Significant difference (day 7 vs. day 12 p<.05).

| **Average Velocity** | **Day 4** | **Day 7** | **Day 12** |
| --- | --- | --- | --- |
| mRNA Axon anterograde (Dim) | .06±.02 | 0.08±.02 | 0.08±.02 |
| mRNA Axon retrograde (Dim) | -0.03±.01 | -0.03±.01 | 0.03±.01 |
| mRNA Dendrite anterograde (Dim) | 0.1±.02 | 0.08±.009 | 0.12±.009 |
| mRNA Dendrite retrograde (Dim) | -0.03±.01 | -0.03±.007 | -0.06±.03 |
| mRNA Axon anterograde (Bright) | 0.005±.001 | 0.009±.001 | 0.004±.001 |
| mRNA Axon retrograde (Bright) | -0.003±.0004 | -0.004±.0005 | -0.004±.0009 |
| mRNA Dendrite anterograde (Bright) | 0.004±.003 | 0.01±.008 | 0.007±.002 |
| mRNA Dendrite retrograde (Bright) | -0.02±.006[*][⯌] | -0.004±.002[⯌] | -0.003±.0008[*] |
| Mitochondria Axon anterograde | 0.003±.0005 | 0.004±.0009 | 0.004±.0005 |
| Mitochondria Axon retrograde | -0.006±.002 | -0.004±.0009 | -0.003±.0005 |
| Mitochondria Dendrite anterograde | 0.001±.0003[⯌] | 0.005±.002[⯌] | 0.002±.0003 |
| Mitochondria Dendrite retrograde | -0.03±.01[*] | -0.03±.01 | -0.03±.01[*] |
