# Supplementary material for: A Comparative Quantitative Assessment of Axonal and Dendritic mRNA Transport in Maturing Hippocampal Neurons
Source: PLoS One. 2013 Jul 22;8(7):e65917. doi: 10.1371/journal.pone.0065917 (PMC3718819; doi:10.1371/journal.pone.0065917)
Supplement: Table S3 — Summary of duration spent moving in each direction for various classes of labeled cargoes. * Significant difference (day 4 vs. day 12 *p<0.05). ⋆ Significant difference (day 4 vs. day 7 ⋆p<0.05). ✶ Significant difference (day 7 vs. day 12 ✶p<0.05). (DOC) [file pone.0065917.s011.doc]

Table S3: Summary of duration spent moving in each direction for various classes of labeled cargoes. * Significant difference (day 4 vs. day 12 p<.05).  Significant difference (day 4 vs. day 7p<.05). Significant difference (day 7 vs. day 12 p<.05).

| **Particle Duration** | **Day 4** | **Day 7** | **Day 12** |
| --- | --- | --- | --- |
| mRNA Axon anterograde (Dim) | 144 ±34[⯌] | 143±17[⯌] | 179±19 |
| mRNA Axon retrograde (Dim) | 84±20 | 100±16 | 83±15 |
| mRNA Dendrite anterograde (Dim) | 110±21 | 200±22 | 168±18 |
| mRNA Dendrite retrograde (Dim) | 67±23 | 61±14 | 27±18 |
| mRNA Axon anterograde (Bright) | 297±34 | 234±26[🟒] | 330±24[🟒] |
| mRNA Axon retrograde (Bright) | 363±40 | 300±34 | 311±36 |
| mRNA Dendrite anterograde (Bright) | 225±50 | 281±32 | 320±31 |
| mRNA Dendrite retrograde (Bright) | 294±86 | 278±71 | 331±56 |
| Mitochondria Axon anterograde | 472±36[*] | 473±37[🟒] | 255±26[*][🟒] |
| Mitochondria Axon retrograde | 308±33[*] | 260±33[🟒] | 517±31[*][🟒] |
| Mitochondria Dendrite anterograde | 264±50[*] | 342±42 | 449±47[*] |
| Mitochondria Dendrite retrograde | 399±49 | 398±42 | 298±48 |
