# Supplementary material for: A Comparative Quantitative Assessment of Axonal and Dendritic mRNA Transport in Maturing Hippocampal Neurons
Source: PLoS One. 2013 Jul 22;8(7):e65917. doi: 10.1371/journal.pone.0065917 (PMC3718819; doi:10.1371/journal.pone.0065917)
Supplement: Table S4 — Summary of net velocities for various classes of labeled cargoes. * Significant difference (day 4 vs. day 12 *p<0.05). ✶ Significant difference (day 4 vs. day 7 ✶p<0.05). (DOC) [file pone.0065917.s012.doc]

Table S4: Summary of net velocities for various classes of labeled cargoes. * Significant difference (day 4 vs. day 12 p<.05). ⯌ Significant difference (day 4 vs. day 7p<.05).
🟒Significant difference (day 7 vs. day 12 p<.05).

| **Net Average Velocity** | **Day 4** | **Day 7** | **Day 12** |
| --- | --- | --- | --- |
| mRNA Axon (Dim) | 0.03±.02 | 0.04±.01 | 0.04±.01 |
| mRNA Dendrite (Dim) | 0.0705±.03 | 0.05±.02 | 0.1±.02 |
| mRNA Axon (Bright) | 0.0002±.0003 | 0.002±.001 | 0.0001±.0004 |
| mRNA Dendrite (Bright) | -0.003±.001[*] | -0.0001±.002 | 0.001±.0006[*] |
| Mitochondria Axon | -0.0002±.0002 | -0.0004±.0007 | 0.0006±.0003 |
| Mitochondria Dendrite | -.002±.0005[*] [⯌] | .0008±.0009[⯌] | .0008±.0004[*] |
