# Supplementary material for: A Comparative Quantitative Assessment of Axonal and Dendritic mRNA Transport in Maturing Hippocampal Neurons
Source: PLoS One. 2013 Jul 22;8(7):e65917. doi: 10.1371/journal.pone.0065917 (PMC3718819; doi:10.1371/journal.pone.0065917)
Supplement: Text S1 — Results related to Mitochondria transport analysis are presented in this section. (DOC) [file pone.0065917.s013.doc]

**Results:**

**Mitochondrial transport in dendrites and axons**

*Maximum and average velocity:* For mitochondria, there was an effect of the type of neurite (p < 0.02), but no effects of the stage of maturity or interaction effect on maximum anterograde velocities. There were also no effects of the type of neurites, stage of maturity, or their interaction on velocities in the retrograde direction. When distributions were compared, in axons, mitochondria revealed significant differences in their maximum velocity between days 4 and 12, and days 7 and 12 in both anterograde and retrograde directions (Fig S7a-b). In dendrites, mitochondria also showed significant differences in the maximum anterograde velocity among all three days. However, in the retrograde direction, significant differences were only found between days 4 and 12 (Fig S7c-d). Though significant, differences in the distributions in both axons and dendrites appear subtle visually, and likely reflect differences in the percentage of mitochondria moving at the slowest velocities (left-most bin).

*Directionality analysis:* Mitochondria displayed a different movement profile than mRNA. In general, mitochondria were less mobile in axons, though there were more moving particles at day 7 (76%) compared to days 4 and 12 (63% and 55%, respectively). Of the unidirectionally moving particles, there were more retrograde particles than anterograde particles at days 4 and 7. Significant differences within a particular population were only found between days 4 and 7; more particles moved anterogradely at day 7 compared to day 4 (p< 0.05), concurrent with a reduction in “wiggling” bidirectional particles (p<0.02; Fig S8a).

In dendrites, there were no significant differences found. However, as was the case for mRNA, there was a strong trend towards increased anterograde mitochondrial transport over time. Between days 4 and 7, this increase occurred concurrently with a decrease in retrograde particles. Between days 7 and 12, both anterograde and retrograde populations increased as stationary populations decreased.

*Duration of directional movements:* Mitochondria, like bright mRNA, increased their duration of anterograde movement in both axons and dendrites at later stages of development*.* Significant differences in the distributions of anterograde durations were found for days 4 vs. 12 (p < 0.0007), and between days 7 and 12 (p < 0.0001) in axons. No significant differences were found in the dendrites. Unlike mRNA, though, in the retrograde direction, distributions of durations of moving mitochondrial mRNA particles differed in axons, with a leftward shift (shorter) at later time points (4 vs. 12, p < 0.0001, and 7 vs. 12 p < 0.001), and in dendrites between days 4 and 12 (p < 0.02).

*Net velocity:* The net velocity of mitochondria illustrated a different pattern from mRNA. In axons, net velocity was retrograde at days 4 and 7, before increasing anterogradely at day 12 (Supplemental Fig 8c). Net velocity also increased over time in dendrites, though in this case a retrograde net velocity was observed at day 4 before changing direction and increasing in magnitude at days 7 and 12, resulting in significant differences among all three days (Fig S8d).

**Discussion:**

**Mitochondrial transport profiles in axons and dendrites**

Mitochondrial transport has been characterized extensively in neurons of both the PNS and CNS. The bulk of this work has been performed in chick sympathetic neurons (e.g., [57,58]), though at least two papers [29,59] have quantified mitochondrial transport in rat hippocampal neurons, and further, axons and dendrites. Consistent with these studies [29,59,60], we observed that individual mitochondria moved with both anterograde and retrograde net directionality in axons and dendrites. The total percentage of directionally moving mitochondria (Fig S7 a-d) was not significantly different in axons and dendrites, and slightly higher than the 20-40% previously observed [29,59,60], though this difference may be a result of our slightly lower cutoff for stationary particles (0.001μm/sec vs. 0.01 μm/sec). In axons, we observed that mitochondria initially demonstrated a slight retrograde net velocity at early stages of growth and synapse formation (days 4 and 7), and an increase in anterograde net velocity as neurons further matured (day 12). These changes were primarily a result of changes in the duration spent moving in a particular direction, rather than differences in the velocity or directionality of individual particles (Fig S7 e,f). In dendrites, mitochondria also displayed a slight retrograde net velocity at day 4 before reversing to a net anterograde velocity at days 7 and 12. In this case, however, differences were a consequence of differing directionalities of individual particles across days (Fig S8d).

The observed differences in mitochondrial transport at varying stages of axonal and dendritic maturity are consistent with previous suggestions that mitochondrial movement is dependent on both the stage of growth and position within a neurite [29,58,59,60]. The specific pattern, though, appears to be heavily dependent on energetic requirements within a particular experimental model. For example, our observed increase in anterograde transport with developmental maturity is opposite to that observed in sympathetic neurons [57,58]**,** which showed net anterograde mitochondrial transport in growing axons, but net retrograde transport in halted axons. This could reflect mitochondrial recruitment for synaptic activity and cytoskeletal stabilization in stationary CNS neurites (Fig 1; [61,62]) but the lack of such recruitment in axons whose growth is truncated by a non-physiological barrier [58]. Mitochondrial transport is also likely to depend heavily on the localization of existing pools of mitochondria. For example, in PNS axons, the highest levels of directional movement occur in regions away from the growth cone [57,58], while a large pool of mitochondria already localizes to the distal axon and growth cone [58]. This is in contrast to our observations of mitochondria more or less evenly distributed along the axon.
